# Synthesis and Biological Evaluation of *NH*_2_-Sulfonyl Oseltamivir Analogues as Influenza Neuraminidase Inhibitors

**DOI:** 10.3390/molecules24112176

**Published:** 2019-06-10

**Authors:** Yaping Hu, Binfeng Chen, Zaiqiang Lei, Hongqian Zhao, Hongxi Zhu, Peng Quan, Yongshou Tian

**Affiliations:** 1 Key Laboratory of Structure-Based Drug Design and Discovery, Ministry of Education, School of Pharmaceutical Engineering, Shenyang Pharmaceutical University, Shenyang 110016, Liaoning, China; hyp910309@163.com (Y.H.); yh_cbf@163.com (B.C.); ZaiqiangLei@126.com (Z.L.); zhaohongqian999@163.com (H.Z.); 13125557362@163.com (H.Z.); 2 School of Pharmacy, Shenyang Pharmaceutical University, Shenyang 110016, Liaoning, China

**Keywords:** influenza, neuraminidase inhibitors, oseltamivir analogues

## Abstract

A series of *NH*_2_-sulfonyl oseltamivir analogues were designed, synthesized, and their inhibitory activities against neuraminidase from H5N1 subtype evaluated. The results indicated that the IC_50_ value of compound **4a**, an oseltamivir analogue via methyl sulfonylation of C5-*NH*_2_, was 3.50 μM. Molecular docking simulations suggested that **4a** retained most of the interactions formed by oseltamivir carboxylate moieties and formed an additional hydrogen bond with the methylsulfonyl group. Meanwhile, **4a** showed high stability towards human liver microsomes. More importantly, **4a** without basic moieties is not a zwitterion as reported on the general structure of neuraminidase inhibitors. This research will provide valuable reference for the research of new types of neuraminidase inhibitors.

## 1. Introduction

Influenza, an infectious disease caused by influenza viruses, has a high morbidity and mortality. In the 20th century, there were three pandemic outbreaks of influenza, for example, the “Spanish” influenza resulting in approximately 50 million deaths in 1918 and 1919 [1]. The pandemic nature of influenza presents a serious public health concern [2,3]. At present, there are two types of agents for influenza, adamantane-based M2 ion channel protein inhibitors and neuraminidase inhibitors (NAIs). The application of the former is limited due to adverse side effects and rapidly developed drug resistance. Thus the latter is widely used [4,5,6,7]. So far, four neuraminidase inhibitors, oseltamivir phosphate [6] and zanamivir hydrate [8] approved around the world, peramivir hydrate [9,10], and laninamivir octanoate [11] marketed only in a few countries, are available. Because of its convenient oral administration, oseltamivir has the largest market share [12,13].

**Figure 1:**
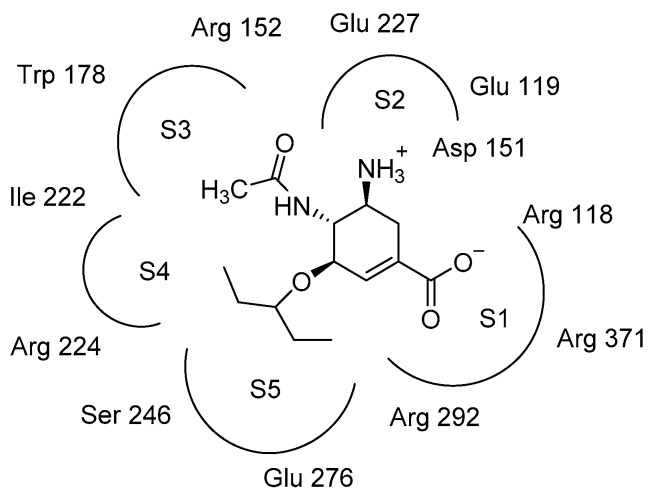
2D diagram of S1–S5 of the active site with oseltamivir carboxylate (OC).

Neuraminidase (NA) existing as a tetramer is responsible for removing sialic acid residues from receptors and facilitates virus release and spread to infect host cells [3,8,14]. The active site of NA is divided into five subsites (S1–S5) (Figure 1). S1, composed of three basic amino acids (Arg118, Arg292, and Arg371), has affinity for the acidic moieties of the inhibitors to form a salt bridge beneficial for potent inhibitory activity. S2, formed by three acidic amino acids (Glu119, Asp151, and Glu227), has affinity for basic moieties to form a salt bridge [7,15]. Therefore, powerful inhibitors are generally zwitterions with poor lipophilicity and oseltamivir carboxylate (OC, the active ingredient of oseltamivir as a prodrug) is no exception [16]. The non-zwitterionic NAIs have potential advantages for several aspects such as lipophilicity.

**Figure 2:**
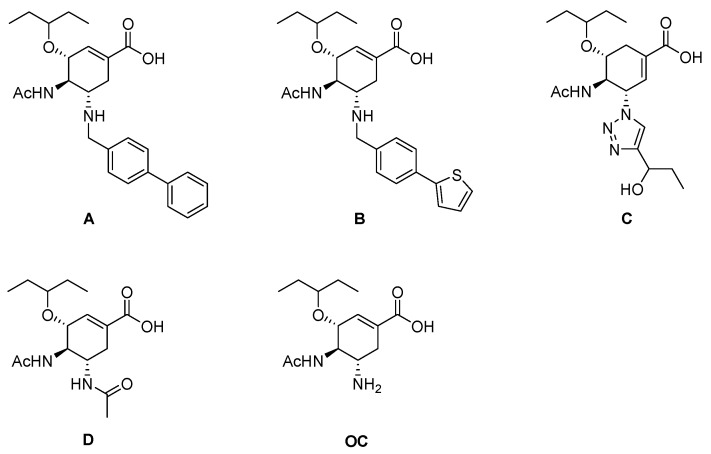
Chemical structures of compound **A**, **B**, **C,** and **D**, OC.

**Figure 3:**
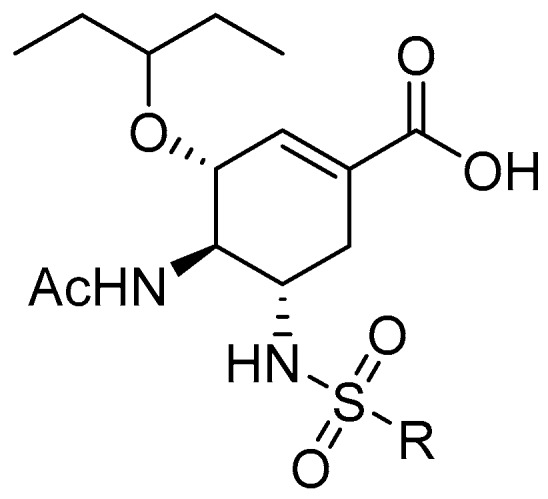
The general structure of designed compounds.

The 150-cavity close to S2, formed by residues of 147–152, offered new opportunities for the research of a new type of NAIs [7,14]. Guided by the 150-cavity, several oseltamivir derivatives substituted on the C5-amine have been reported. Xie and his colleagues revealed compounds **A** and **B** (Figure 2) with IC_50_ values of 1.9 nM and 2.1 nM against NAs from H5N1 (A/Chicken/China/1220/2012) respectively, which preliminarily showed that the 150-cavity was an advantage for enhancing inhibitory activity [17]. In 2010, Mohan et al. discovered compound **C** containing triazole with a *K_i_* value of 72 nM. Saturation transfer difference (STD) NMR spectroscopic studies suggested that compound **C** interacted with the 150-cavity successfully [12]. Our laboratory discovered compound **D** (Figure 2) which showed powerful inhibitory activities against wild-type NAs with IC_50_ values similar to those of OC and enhanced inhibitory activities against mutant NAs in the process of searching for inhibitors targeting the 150-cavity. What is more, compound **D** without basic moieties was not consistent with the traditional structure–activity relationships in that a basic moiety is essential for powerful activity [18]. In consideration that sulfonamido (R^1^SO_2_NHR^2^) can act both as hydrogen bond donor and hydrogen bond acceptor, which may be beneficial for biological activity [19,20], we continue to search for NAIs without basic moieties. A series of oseltamivir analogues bearing sulfonamido were designed with the aim of the sulfonamido generating hydrogen bonds with the S2 and R (Figure 3) group interacting with the 150-cavity.

## 2. Results and Discussion

### 2.1. Synthesis

**Scheme 1:**
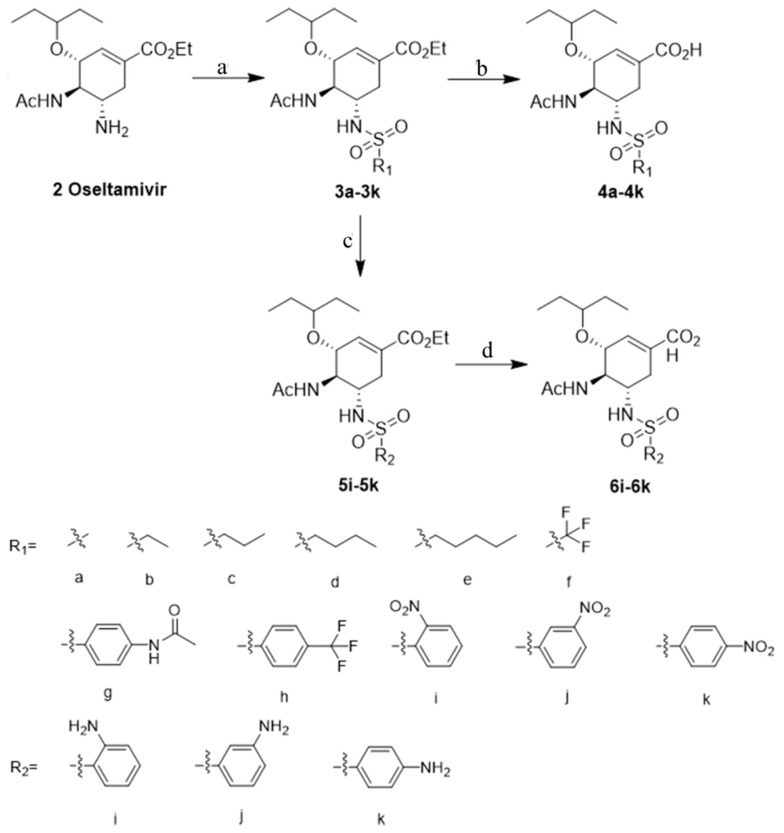
Synthetic route of target compounds (**4a**–**4k** and **6i**–**6k**): Reagents and conditions: (**a**) corresponding sulfonyl chloride, TEA, CH_2_Cl_2_, 0 °C; (**b**) NaOH, CH_3_OH/H_2_O; (**c**) Fe, NH_4_Cl, 90% CH_3_CH_2_OH/H_2_O; (**d**) NaOH, CH_3_OH/H_2_O.

The synthetic approaches to oseltamivir were investigated, and several approaches were found to be innovative or interesting [21,22,23,24], however, we followed our previous semi-synthetic procedure for oseltamivir (**2**) [18]. The synthetic route of the target compounds is depicted in Scheme 1. Oseltamivir was reacted with the corresponding sulfonyl chlorides to obtain **3a**–**3k** [18,25,26]. The nitro fragment of **3i**–**3k** was reduced to amino by iron powder to afford intermediates **5i**–**5k**. Finally, **3a**–**3k** and **5i**–**5k** were treated with NaOH in aq. methanol to yield target compounds **4a**–**4k** and **6i**–**6k** via saponification. All compounds were confirmed by ^1^H-NMR, ^13^C-NMR, and HRMS (ESI).

### 2.2. Neuraminidase Enzyme Inhibitory Assay

Compounds **4a**–**4k** and **6i**–**6k** were evaluated as inhibitors of the NA from the H5N1 subtype of influenza A. The inhibition rates at 10 μM and 100 μM are shown in Table 1 [18]. The relatively good compounds such as compound **4a**, **4h**, **4i,** and **6i** were further screened to give IC_50_ values. The inhibitory activities varied from the sulfonyl fragments substituted on the amine of OC. Compounds **4a**–**4e** showed high to weak inhibitory activities. The length of the sulfonyl moieties played a crucial role in the inhibitory activities. The increased length of the substituents led to decreased inhibitions against NA as suggested by **4a**–**4e**. Compound **4a,** possessing the shortest substituent, exhibited the most powerful neuraminidase inhibitory activity with an IC_50_ value of 3.50 μM (Table 2). Meanwhile, **4f** as a fluoro-substituted congener of **4a** did not exhibit good inhibitory activity, indicating fluorine substitution was harmful for interaction with amino acids near or belonging to S2. Compounds **4g**–**4k** and **6i**–**6k** bearing aromatic rings exhibited moderate to weak inhibitory activities. Compound **4g** containing 4-acetylamido phenyl exerted little inhibition even at 100 μM. Compared with **4f**, the inhibitory activity of compound **4h** bearing trifluoromethyl was enhanced, and the IC_50_ value was 12.00 μM. Among the compounds **4i**–**4k** and **6i**–**6k**, *ortho*-substituted **4i** and **6i** showed more potent inhibitory activities than the *meta*-substituted and *para*-substituted counterparts. Inhibitory activities decreased in the order of *ortho*-, *meta*-, and *para*-position. Compared to the nitro, the amino group was more beneficial for inhibitory activity.

Compound **4a** exerted the most powerful inhibitory activity. The inhibition of **4a** was weaker than that of OC, but **4a** without basic moieties is not a zwitterion, which is not consistent with traditional structure–activity relationships of NAIs.

### 2.3. Molecular Docking Model Analysis

**Figure 4:**
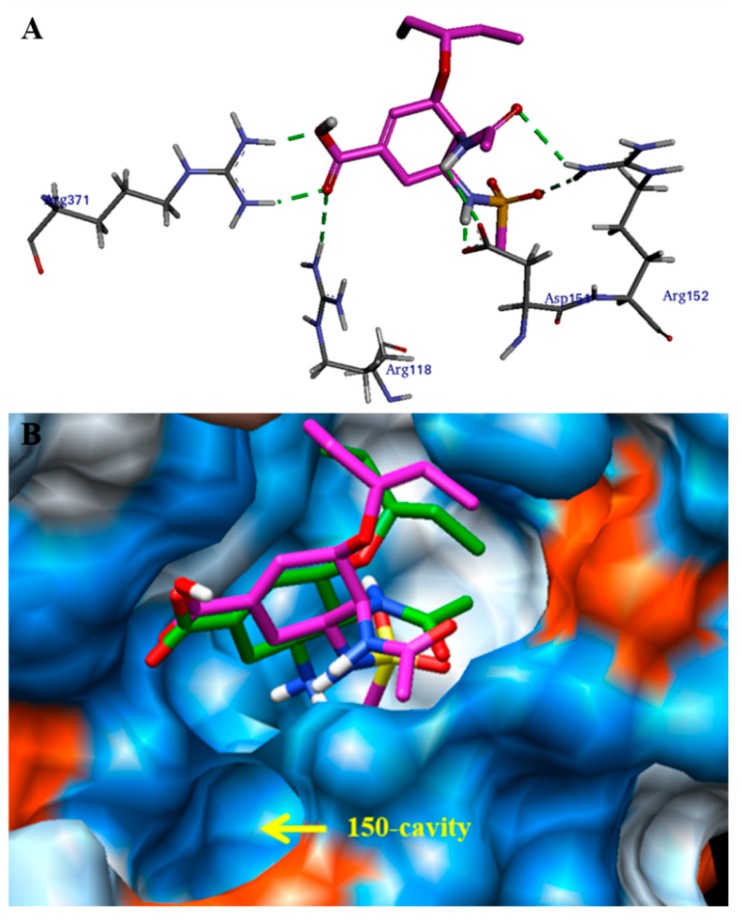
(**A**) The docking modes of compound **4a** (purple) with NA from H5N1 (PDB code 2HU4, downloaded from RCSB PDB (http://www.rcsb.org/). This figure was done by Glide program in MAESTRO software. (**B**) Molecular surface representation of the neuraminidase active site with OC (green) and **4a** (purple), and the 150-cavity indicated. This figure was carried out by Chimera.

As shown in Figure 4B, due to the added methylsulfonyl group the OC fragment of compound **4a** did not overlap well with OC, so the interactions formed by the OC moiety with NA were discounted to a certain extent. It is a pity that on account of the short length of the methylsulfonyl compound **4a** could not access the 150 cavity as expected. The oxygen of the sulfonyl of compound **4a** generated a new hydrogen bond with Arg152 (Figure 4A). The new interactions generated by methylsulfonyl were not enough to make up for the lost or decreased ones formed by the C5 amine of the OC fragment with S2, which explained the weaker inhibitory activity of **4a** compared to that of OC.

### 2.4. Metabolic Stability in Human Liver Microsomes In Vitro

Compound **4a** with the most powerful inhibition was selected to test the metabolic stability in the presence of human liver microsomes in vitro and the control compounds of testosterone, diclofenac, and propafenone validated the assay [27,28,29,30]. The results are shown in Table 3. After incubating with human liver microsomes for 1 h, almost of all of **4a** (101.6%) was detected and the **T_1/2_** (half time) was more than 145 min, which indicated that **4a** showed high stability towards human liver microsomes in vitro.

## 3. Materials and Methods

### 3.1. Chemistry

All of the raw materials and solvents were purchased from commercial suppliers. Melting points were determined in slides on a WRX-4 Micro melting point apparatus (Yice). High resolution mass spectra (HRMS) were recorded on an Agilent 6530 ultrahigh definition (UHD) accurate mass Q-TOF MS by ESI mode. The 1H-nuclear magnetic resonance (NMR) and ^13^C-NMR spectra were recorded on a Bruker ARX 600 MHz using tetramethylsilane as the internal standard. The reaction process was monitored by thin-layer chromatography (TLC) on silica gel GF254. Plates were visualized using UV light (254 nm). The purity of target compound was determined by HPLC. The detailed method is as follows: a Shimadzu (Kyoto, Japan) HPLC; Column: DIAMONSIL^®^ C18, 250 mm × 4.6mm, 5 μm; mobile phase (isocratic elution): 50% acetonitrile (0.1% trifluoroacetic acid) for method A; 35% acetonitrile (0.1% trifluoroacetic acid) for method B; 25% acetonitrile (0.1% trifluoroacetic acid) for method C. Flow rate: 1 mL/min; Detector: UV (254 nm).

#### General Procedure for the Preparation of Compounds **4a**–**4k** and **6i**–**6k**

Oseltamivir (**2**, 312.4 mg, 1.0 mmol), TEA (208 μL, 1.5 mmol) and 10 mL CH_2_Cl_2_ were charged in a 50 mL round bottom flask, then the corresponding sulfonyl chloride (1.2 mmol) was added dropwise. The mixture was stirred at 0 °C until oseltamivir was completely consumed, as indicated by TLC analysis. The organic layer was washed successively with 1N HCl aqueous solution, saturated aq.Na_2_CO_3,_ and brine. The organic layer was concentrated in vacuo. The crude product was purified by column chromatography to obtain one of the intermediates (**3a**–**3k**). 

One of intermediates **3a**–**3k** (1.0 mmol), 1N NaOH aqueous solution (2.5 mmol), methanol (15 mL) and deionized water (*V* (methanol): *V* (water) = 5:1) were added to a round bottom flask. The mixture was stirred at room temperature. Then the methanol was evaporated in vacuo and the residual solution was acidified with 1N HCl aqueous solution to pH 1 to 2. The precipitate was separated and filtered. Finally, one of the title compounds (**4a**–**4k**) was obtained. 

A mixture of one of compounds (**3i**–**3k**) (1 mmol), iron powder (8 mmol) and NH_4_Cl (10 mmol) in 90% ethanol aqueous solution (30 mL) was stirred under reflux until the starting material was consumed completely, as indicated by TLC analysis. The precipitate was filtered, and the filtrate was evaporated in vacuo. The residue was extracted by dichloromethane until the new dichloromethane did not contain one of intermediates (**5i**–**5k**) any more, and the organic layer was concentrated in vacuo to obtain one of the crude compounds (**5i**–**5k**). Following the procedure for compounds **4a**–**4k**, the compounds (**6i**–**6k**) were obtained. More detailed information can be found in the supplementary materials.

*(3R, 4R, 5S)-4-acetamido-5-(methylsulfonyl)amino-3-(pentan-3-yloxy) cyclohex-1-enecarboxylic acid* (**4a**). White solid, m.p. 212.9–215.2 °C, yield, 45%; ^1^H-NMR (600MHz, DMSO-*d*_6_): δ 12.61 (s, 1H), 7.86 (d, *J* = 9.2 Hz, 1H), 7.04 (d, *J* = 9.0 Hz, 1H), 6.60 (s, 1H), 4.11 (d, *J* = 8.7 Hz, 1H), 3.63 (dd, *J* = 20.2, 9.1 Hz, 1H), 3.36 (dt, *J* = 10.9, 5.6 Hz, 2H), 2.90 (s, 3H), 2.64 (dd, *J* = 17.5, 5.4 Hz, 1H), 2.28–2.22 (m, 1H), 1.84 (s, 3H), 1.45–1.35 (m, 4H), 0.84 (t, *J* = 7.4 Hz, 3H), 0.79 (t, *J* = 7.4 Hz, 3H); ^13^C-NMR (150 MHz, DMSO-*d*_6_): δ 170.10, 167.58, 138.09, 129.36, 81.45, 75.66, 54.20, 52.46, 41.88, 32.88, 26.17, 25.59, 23.46, 9.96, 9.38; ESI-HRMS (*m/z*), Calcd. for C_15_H_26_N_2_O_6_S[M − H]^−^: 361.1439, found: 361.1452. Method C: retention time: 8.64 min, 96.7% purity.

*(3R, 4R, 5S)-4-acetamido-5-(ethylsulfonyl)amino-3-(pentan-3-yloxy)cyclohex-1-enecarboxylic acid* (**4b**). White solid, m.p. 187.7–190.0 °C, yield, 50%; 1H-NMR (600 MHz, DMSO-*d*_6_): δ 12.58 (s, 1H), 7.84 (d, *J* = 9.2 Hz, 1H), 7.00 (d, *J* = 9.2 Hz, 1H), 6.59 (s, 1H), 4.09 (d, *J* = 8.7 Hz, 1H), 3.63 (dd, *J* = 20.0, 9.2 Hz, 1H), 3.37–3.34 (m, 1H), 2.98 (q, *J* = 7.1 Hz, 2H), 2.63 (dd, *J* = 18.2, 5.8 Hz, 1H), 2.30–2.23 (m, 1H), 2.02–1.95 (m, 1H), 1.83 (s, 3H), 1.41 (ddd, *J* = 20.5, 13.0, 6.7 Hz, 4H), 1.17 (t, *J* = 7.3 Hz, 3H), 0.84 (t, *J* = 7.4 Hz, 3H), 0.79 (t, *J* = 7.4 Hz, 3H); ^13^C-NMR (150 MHz, DMSO-*d*_6_): δ 170.00, 167.58, 138.09, 129.34, 81.41, 75.73, 54.24, 52.29, 47.36, 33.11, 26.14, 25.55, 23.53, 9.97, 9.34, 8.65; ESI-HRMS (*m/z*), Calcd. for C_16_H_28_N_2_O_6_S[M − H]^−^: 375.1590, found: 375.1618. Method B: retention time: 5.05 min, 99.4% purity

*(3R, 4R, 5S)-4-acetamido-5-(n-propylsulfonyl)amino-3-(pentan-3-yloxy)cyclohex-1-enecarboxylic acid* (**4c**). White solid, m.p. 196.3–198.6 °C, yield, 70%; ^1^H-NMR (600 MHz, DMSO-*d*_6_): δ 12.64 (s, 1H), 7.84 (d, *J* = 9.2 Hz, 1H), 6.99 (d, *J* = 9.2 Hz, 1H), 6.58 (s, 1H), 4.08 (d, *J* = 8.5 Hz, 1H), 3.63 (dd, *J* = 20.2, 9.1 Hz, 1H), 3.39–3.34 (m, 1H), 2.95 (tt, *J* = 7.3, 3.7 Hz, 2H), 2.63 (dd, *J* = 17.7, 5.4 Hz, 1H), 2.26 (ddt, *J* = 16.5, 10.2, 3.0 Hz, 1H), 1.82 (s, 3H), 1.68–1.60 (m, 2H), 1.49–1.34 (m, 4H), 0.97 (t, *J* = 7.5 Hz, 3H), 0.83 (t, *J* = 7.4 Hz, 3H), 0.79 (t, *J* = 7.4 Hz, 3H); ^13^C-NMR (150 MHz, DMSO-*d*_6_): δ 169.96, 167.77, 137.78, 129.64, 81.41, 75.78, 54.75, 54.21, 52.38, 33.19, 26.15, 25.57, 23.48, 17.51, 13.18, 9.97, 9.37; ESI-HRMS (*m/z*), Calcd. for C_17_H_30_N_2_O_6_S[M − H]^−^: 389.1746, found: 389.1737. Method B: retention time: 7.05 min, 98.5% purity.

*(3R, 4R, 5S)-4-acetamido-5-(n-butylsulfonyl)amino-3-(pentan-3-yloxy) cyclohex-1-enecarboxylic acid* (**4d**). White solid, m.p. 183.3–184.4 °C, yield, 55%; ^1^H-NMR (600 MHz, DMSO-*d*_6_): δ 12.60 (s, 1H), 7.88 (d, *J* = 9.2 Hz, 1H), 6.99 (d, *J* = 9.2 Hz, 1H), 6.59 (s, 1H), 4.09 (d, *J* = 8.6 Hz, 1H), 3.64 (dd, *J* = 20.1, 9.0 Hz, 1H), 3.36 (dt, *J* = 10.8, 5.4 Hz, 2H), 3.03–2.91 (m, 2H), 2.64 (dd, *J* = 17.6, 5.3 Hz, 1H), 2.30–2.21 (m, 1H), 1.82 (s, 3H), 1.63–1.56 (m, 2H), 1.48–1.41 (m, 2H), 1.41–1.33 (m, 4H), 0.91–0.86 (m, 3H), 0.83 (t, *J* = 7.4 Hz, 3H), 0.79 (t, *J* = 7.4 Hz, 3H); ^13^C-NMR (150 MHz, DMSO-*d*_6_): δ 169.97, 167.55, 138.06, 129.33, 81.41, 75.71, 54.13, 52.77, 52.34, 33.18, 26.15, 25.87, 25.57, 23.45, 21.37, 14.05, 9.95, 9.36; ESI-HRMS (*m/z*), Calcd. for C_18_H_32_N_2_O_6_S[M − H]^−^: 403.1903, found: 403.1932. Method B: retention time: 10.67 min, 99.5% purity.

*(3R, 4R, 5S)-4-acetamido-5-(n-pentylsulfonyl)amino-3-(pentan-3-yloxy)cyclohex-1-enecarboxylic acid* (**4e**). White solid, m.p. 179.1–182.8 °C, yield, 55%; ^1^H-NMR (600 MHz, DMSO-*d*_6_): δ 12.60 (s, 1H), 7.84 (d, *J* = 9.2 Hz, 1H), 6.99 (d, *J* = 9.2 Hz, 1H), 6.59 (s, 1H), 4.08 (d, *J* = 8.7 Hz, 1H), 3.63 (dd, *J* = 20.1, 9.1 Hz, 1H), 3.39–3.34 (m, 1H), 3.02–2.90 (m, 2H), 2.63 (dd, *J* = 17.7, 5.4 Hz, 1H), 2.29–2.22 (m, 1H), 1.82 (s, 3H), 1.66–1.58 (m, 2H), 1.49–1.36 (m, 4H), 1.33 (dd, *J* = 13.9, 7.1 Hz, 2H), 1.31–1.26 (m, 2H), 0.87 (t, *J* = 7.1 Hz, 3H), 0.83 (t, *J* = 7.4 Hz, 3H), 0.79 (t, *J* = 7.4 Hz, 3H); ^13^C-NMR (150 MHz, DMSO-*d*_6_): δ 169.94, 167.58, 138.05, 129.35, 81.43, 75.72, 54.19, 53.08, 52.30, 33.08, 30.29, 26.14, 25.57, 23.52, 23.46, 22.23, 14.21, 9.94, 9.36; ESI-HRMS (*m/z*), Calcd. for C_19_H_34_N_2_O_6_S[M − H]^−^: 417.2059, found: 417.2032. Method A: retention time: 7.12 min, 95.7% purity.

*(3R, 4R, 5S)-4-acetamido-5-((trifluoromethyl)sulfonyl)amino-3-(pentan-3-yloxy)cyclohex-1-enecarboxylic acid* (**4f**). White solid, m.p. 193.1–195.4 °C, yield, 69%; ^1^H-NMR (600 MHz, DMSO-*d*_6_): δ 12.72 (s, 1H), 9.57 (d, *J* = 9.0 Hz, 1H), 7.93 (d, *J* = 9.4 Hz, 1H), 6.60 (s, 1H), 4.16 (d, *J* = 8.8 Hz, 1H), 3.76 (dd, *J* = 20.4, 9.2 Hz, 1H), 3.45–3.40 (m, 1H), 3.38 (dd, *J* = 11.1, 5.6 Hz, 1H), 2.59 (dd, *J* = 17.5, 5.6 Hz, 1H), 2.39–2.32 (m, 1H), 1.84 (s, 3H), 1.49–1.33 (m, 4H), 0.83 (t, *J* = 7.4 Hz, 3H), 0.77 (t, *J* = 7.4 Hz, 3H); ^13^C-NMR (150 MHz, DMSO-*d*_6_): δ 169.88, 167.31, 138.49, 128.48, 119.97 (q, *J* = 321.5 Hz), 81.71, 75.15, 54.54, 54.10, 31.64, 26.18, 25.59, 23.29, 9.87, 9.41; ESI-HRMS (*m/z*), Calcd. for C_15_H_23_F_3_N_2_O_6_S[M − H]^−^: 415.1151, found: 415.1172. Method B: retention time: 16.50 min, 96.0% purity.

*(3R, 4R, 5S)-4-acetamido-5-((4-(acetamido)pheny)sulfonyl)amino-3-(pentan-3-yloxy)cyclohex-1-enecarboxylic acid* (**4g**). White solid, m.p. 236.7–238.6 °C, yield, 60%; ^1^H-NMR (600 MHz, DMSO-*d*_6_): δ 12.51 (s, 1H), 10.32 (s, 1H), 7.74 (d, *J* = 8.8 Hz, 2H), 7.71–7.66 (m, 3H), 7.49 (d, *J* = 8.6 Hz, 1H), 6.53 (s, 1H), 4.06 (d, *J* = 8.4 Hz, 1H), 3.62 (dd, *J* = 19.9, 9.0 Hz, 1H), 3.25–3.18 (m, 1H), 2.25 (dd, *J* = 17.8, 5.4 Hz, 1H), 2.12 (dd, *J* = 10.4, 7.5 Hz, 1H), 2.08 (s, 3H), 1.69 (s, 3H), 1.46–1.29 (m, 4H), 0.81 (t, *J* = 7.4 Hz, 3H), 0.75 (t, *J* = 7.4 Hz, 3H); ^13^C-NMR (150 MHz, DMSO-*d*_6_): δ 170.13, 169.44, 167.43, 143.01, 138.15, 136.25, 129.00, 127.87 (2C), 118.93 (2C), 81.49, 75.28, 54.12, 52.79, 31.71, 26.18, 25.61, 24.63, 23.36, 9.87, 9.46; ESI-HRMS (*m/z*), Calcd. for C_22_H_31_N_3_O_7_S[M − H]^−^: 480.1804, found: 480.1838. Method B: retention time: 6.23 min, 99.2% purity.

*(3R, 4R, 5S)-4-acetamido-5-((4-(trifluoromethyl)phenyl)sulfonyl)amino-3-(pentan-3-yloxy)cyclohex-1-enecarboxylic acid* (**4h**). White solid, m.p. 211.9–213.6 °C, yield, 77%; ^1^H-NMR (600 MHz, DMSO-*d*_6_): δ 12.56 (s, 1H), 7.99 (d, *J* = 9.0 Hz, 4H), 7.66 (d, *J* = 9.1 Hz, 1H), 6.50 (s, 1H), 4.06 (d, *J* = 8.4 Hz, 1H), 3.62 (dd, *J* = 20.0, 9.0 Hz, 1H), 3.33 (dd, 1H), 2.27 (dd, *J* = 17.5, 5.5 Hz, 1H), 2.17–2.09 (m, 1H), 1.90 (s, 1H), 1.60 (s, 3H), 1.41–1.32 (m, 4H), 0.81 (t, *J* = 7.4 Hz, 3H), 0.75 (t, *J* = 7.4 Hz, 3H); ^13^C-NMR (150 MHz, DMSO-*d*_6_): δ 169.95, 146.75, 132.27 (q, *J* = 32.3 Hz,), 127.64 (2C), 126.86 (d, *J* = 3.6 Hz, 2C), 126.76, 124.95, 123.15, 121.34, 81.41, 75.45, 54.29, 53.20, 32.34, 26.19, 25.62, 23.14, 9.86, 9.42; ESI-HRMS (*m/z*), Calcd. for C_21_H_27_F_3_N_2_O_6_S[M − H]^−^: 491.1464, found: 491.1464. Method A: retention time: 8.21 min, 98.6% purity.

*(3R, 4R, 5S)-4-acetamido-5-((2-nitrophenyl)sulfonyl)amino-3-(pentan-3-yloxy)cyclohex-1-enecarboxylic acid* (**4i**). White solid, m.p. 183.6–185.1 °C, yield, 70%; ^1^H-NMR (600 MHz, DMSO-*d*_6_): δ 12.58 (s, 1H), 8.03–8.00 (m, 1H), 7.97–7.94 (m, 2H), 7.89–7.83 (m, 2H), 7.73 (d, *J* = 9.1 Hz, 1H), 6.58 (s, 1H), 4.10 (d, *J* = 8.4 Hz, 1H), 3.71 (dd, *J* = 19.8, 9.0 Hz, 1H), 3.46 (ddd, *J* = 16.5, 10.4, 5.7 Hz, 1H), 3.38–3.34 (m, 1H), 2.40 (dd, *J* = 17.5, 5.5 Hz, 1H), 2.28–2.21 (m, 2H), 1.64 (s, 3H), 1.43–1.35 (m, 4H), 0.82 (t, *J* = 7.4 Hz, 3H), 0.75 (t, *J* = 7.4 Hz, 3H); ^13^C-NMR (150 MHz, DMSO-*d*_6_): δ 170.00, 167.40, 147.59, 138.11, 134.58, 134.33, 133.15, 130.23, 128.94, 124.66, 81.54, 75.17, 53.91, 53.19, 31.61, 26.16, 25.60, 23.18, 9.88, 9.42. ESI-HRMS (*m/z*), Calcd. for C_20_H_27_N_3_O_8_S[M − H]^−^: 468.1441, found: 468.1458. Method A: Retention time: 6.45 min, 99.4% purity.

*(3R, 4R, 5S)-4-acetamido-5-((3-nitrophenyl)sulfonyl)amino-3-(pentan-3-yloxy)cyclohex-1-enecarboxylic acid* (**4j**). Faint yellow solid, m.p. 114.3–115.9 °C, yield, 60%; ^1^H-NMR (600 MHz, DMSO-*d*_6_) δ 8.50 (t, *J* = 1.8 Hz, 1H), 8.47 (dd, *J* = 8.2, 1.5 Hz, 1H), 8.19 (d, *J* = 7.9 Hz, 1H), 8.11 (s, 1H), 7.90 (t, *J* = 8.0 Hz, 1H), 7.66 (d, *J* = 9.1 Hz, 1H), 6.48 (s, 1H), 4.04 (d, *J* = 8.2 Hz, 1H), 3.62 (dd, *J* = 19.9, 9.2 Hz, 1H), 3.33–3.30 (m, 1H), 2.64 (dd, *J* = 13.8, 6.7 Hz, 1H), 2.30 (dd, *J* = 17.7, 5.2 Hz, 1H), 2.16–2.09 (m, 1H), 1.44–1.29 (m, 4H), 1.01 (t, *J* = 7.2 Hz, 3H), 0.81 (t, *J* = 7.4 Hz, 3H), 0.74 (t, *J* = 7.4 Hz, 3H); ^13^C-NMR (150 MHz, DMSO-*d*_6_): δ 169.94, 167.35, 148.24, 144.36, 138.15, 132.77, 131.81, 128.88, 127.27, 121.60, 81.51, 75.32, 54.07, 52.85, 31.94, 26.13, 25.59, 23.19, 9.84, 9.41; ESI-HRMS (*m/z*), Calcd. for C_20_H_27_N_3_O_8_S[M − H]^−^: 468.1441, found: 468.1470. Method A: retention time: 7.00 min, 99.3% purity.

*(3R, 4R, 5S)-4-acetamido-5-((4-nitrophenyl)sulfonyl)amino-3-(pentan-3-yloxy)cyclohex-1-enecarboxylic acid* (**4k**). White solid, m.p. 187.4–189.2 °C, yield, 55%; ^1^H-NMR (600 MHz, DMSO-*d*_6_): δ 12.57 (s, 1H), 8.41 (d, *J* = 8.8 Hz, 2H), 8.12 (d, *J* = 8.8 Hz, 1H), 8.03 (d, *J* = 8.8 Hz, 2H), 7.69 (d, *J* = 9.1 Hz, 1H), 6.54 (s, 1H), 4.07 (d, *J* = 8.5 Hz, 1H), 3.64 (dd, *J* = 20.0, 9.0 Hz, 1H), 3.39–3.34 (m, 1H), 2.29 (dd, *J* = 17.6, 5.4 Hz, 1H), 2.19–2.11 (m, 1H), 1.64 (s, 3H), 1.46–1.29 (m, 4H), 0.81 (t, *J* = 7.4 Hz, 3H), 0.75 (t, *J* = 7.4 Hz, 3H); ^13^C-NMR (150 MHz, DMSO-*d*_6_): δ 170.03, 167.34, 149.74, 148.26, 138.15, 128.89, 128.35, 125.00, 81.48, 75.22, 54.15, 53.02, 31.95, 26.16, 25.61, 23.24, 9.85, 9.42; ESI-HRMS (*m/z*), Calcd. for C_20_H_27_N_3_O_8_S[M − H]^−^: 468.1441, found: 468.1456. Method A: retention time: 7.22 min, 99.8% purity.

*(3R, 4R, 5S)-4-acetamido-5-((2-aminophenyl)sulfonyl)amino-3-(pentan-3-yloxy)cyclohex-1-enecarboxylic acid* (**6i**). White solid, m.p. 161.8–164.0 °C, yield, 58%; ^1^H-NMR (600 MHz, DMSO-*d*_6_): δ 12.28 (s, 1H), 7.65 (d, *J* = 9.2 Hz, 1H), 7.54–7.44 (m, 2H), 7.27–7.20 (m, 1H), 6.79 (d, *J* = 8.2 Hz, 1H), 6.58 (t, *J* = 7.5 Hz, 1H), 5.88 (s, 2H), 4.06 (d, *J* = 8.2 Hz, 1H), 3.62 (dd, *J* = 19.9, 9.1 Hz, 1H), 3.34 (s, 1H), 3.11 (s, 1H), 2.29 (dd, *J* = 17.7, 5.2 Hz, 1H), 2.15–2.05 (m, 1H), 1.90 (s, 3H), 1.78 (s, 3H), 1.46–1.30 (m, 4H), 0.81 (t, *J* = 7.4 Hz, 3H), 0.76 (t, *J* = 7.4 Hz, 3H); ^13^C-NMR (150 MHz, DMSO-*d*_6_): δ 170.20, 167.43, 146.52, 138.30, 133.85, 129.34, 129.02, 121.39, 117.21, 115.37, 81.51, 75.30, 54.13, 52.20, 31.43, 26.16, 25.61, 23.47, 9.87, 9.45. ESI-HRMS (*m/z*), Calcd. for C_20_H_29_N_3_O_6_S[M − H]^−^: 438.1699, found: 438.1711. Method A: retention time: 5.99 min, 98.9% purity.

*(3R, 4R, 5S)-4-acetamido-5-((3-aminophenyl)sulfonyl)amino-3-(pentan-3-yloxy)cyclohex-1-enecarboxylic acid* (**6j**). White solid, m.p. 177.4–178.1 °C, yield, 40%; ^1^H-NMR (600 MHz, DMSO-*d*_6_): δ 7.25 (d, *J* = 7.7 Hz, 1H), 7.16 (t, *J* = 7.9 Hz, 1H), 6.94 (t, *J* = 2.0 Hz, 1H), 6.86 (dd, *J* = 4.6, 3.8 Hz, 1H), 6.72 (ddd, *J* = 8.1, 2.2, 0.8 Hz, 1H), 6.30 (s, 1H), 5.52 (s, 1H), 4.01 (d, *J* = 8.1 Hz, 2H), 3.59 (dd, *J* = 19.9, 8.9 Hz, 1H), 3.46–3.40 (m, 1H), 3.13 (s, 1H), 2.31 (dd, *J* = 17.7, 5.2 Hz, 1H), 2.10–2.03 (m, 1H), 1.74 (s,3H), 1.46–1.28 (m, 4H), 0.81 (t, *J* = 7.4 Hz, 3H), 0.75 (t, *J* = 7.4 Hz, 3H).; ^13^C-NMR (150 MHz, DMSO-*d*_6_): δ 170.43, 168.63, 149.70, 143.05, 135.11, 131.90, 129.89, 117.47, 113.54, 111.29, 81.32, 75.61, 54.52, 53.38, 32.39, 26.29, 25.68, 23.35, 9.86, 9.51. ESI-HRMS (*m/z*), Calcd. for C_20_H_29_N_3_O_6_S[M − H]^−^: 438.1699, found: 438.1713. Retention time: 4.0 min, 99.2% purity.

*(3R, 4R, 5S)-4-acetamido-5-((4-aminophenyl)sulfonyl)amino-3-(pentan-3-yloxy)cyclohex-1-enecarboxylic acid* (**6k**). Faint yellow solid, m.p. 176.7–181.4 °C, yield, 40%; ^1^H-NMR (600 MHz, DMSO-*d*_6_): δ 7.63 (d, *J* = 9.1 Hz, 1H), 7.38 (d, *J* = 8.7 Hz, 2H), 6.97 (d, *J* = 8.2 Hz, 1H), 6.58 (d, *J* = 8.7 Hz, 2H), 6.37 (s, 1H), 5.89 (s, 2H), 4.01 (d, *J* = 8.1 Hz, 1H), 3.62–3.54 (m, 1H), 3.33 (dt, *J* = 11.2, 5.6 Hz, 1H), 3.10–3.03 (m, 1H), 2.32 (dd, *J* = 17.8, 5.5 Hz, 1H), 2.06 (ddd, *J* = 10.2, 8.7, 5.0 Hz, 1H), 1.74 (s, 3H), 1.46–1.28 (m, 4H), 0.81 (t, *J* = 7.4 Hz, 3H), 0.75 (t, *J* = 7.4 Hz, 3H); ^13^C-NMR (150 MHz, DMSO-*d*_6_): δ 170.25, 167.50, 152.78, 138.07, 129.15, 128.69 (2C), 127.45, 113.08 (2C), 81.47, 75.26, 52.60, 46.09, 31.67, 26.20, 25.63, 23.41, 9.86, 9.48; ESI-HRMS (*m/z*), Calcd. for C_20_H_29_N_3_O_6_S[M − H]^−^: 438.1699, found: 438.1714. Method B: retention time: 5.54 min, 97.9% purity.

### 3.2. Biological Evaluation

#### 3.2.1. Neuraminidase Enzyme Inhibitory Assay

The H5N1 neuraminidase (A/Anhui/2005) was purchased from Sino Biological Inc (Beijing, China). OC as a positive control was purchased from MedChemExpress (Monmouth Junction, NJ, USA). Fluorogenic substrate MUNANA (2′-(4-methylumbelliferyl)-α-*N*-acetylneuraminic acid) was purchased from Sigma-Aldrich (Munich, Germany). The procedure followed previous method [19].

#### 3.2.2. Metabolic Stability Assay

The human liver microsomes were purchased from BD (San Jose, USA). NADPH was purchased from Sigma-Aldrich (Munich, Germany). Two parallel assays with and without NADPH regenerating system were determined.

The compound **4a** and control compounds (testosterone, diclofenac, or propafenone) were prepared by dilution of reaction buffer, and the final concentration of human liver microsomes was 0.5 mg/mL. Incubation was carried out in a thermostat at 37 °C and started by the addition of the appropriate compound. The samples were taken at 0, 5, 10, 20, 30, and 60 min. The reaction was terminated by the addition of stop solution. After collection, samples were centrifuged (20 min, 4000 rpm), then the centrifuged supernatant was directly analyzed by using LC-MS/MS analysis. Metabolic half-time (T_1/2_) was calculated by using the equation of first order kinetics.

## 4. Conclusions

In summary, a series of oseltamivir analogues bearing the sulfonamido group were designed based on our previous work on potent neuraminidase inhibitors without basic moieties and with the opportunities offered by the 150-cavity. Among these analogues, compound **4a** showed the most potent inhibition against NA from H5N1 subtype with an IC_50_ value of 3500 nM. Molecular docking simulations revealed that **4a** retained most of the interactions formed by the OC fragment and generated a new hydrogen bond. Due to the short length of the methylsulfonyl group, **4a** did not get access to the 150-cavity as expected. Meanwhile, **4a** exhibited high metabolic stability against human liver microsomes in vitro. What is more, compound **4a** without basic moieties is not consistent with traditional inhibitors as zwitterions. Thus this study has enriched the structure types of NAIs and may provide valuable reference for the discovery of new types of NAIs.

## Figures and Tables

**Table 1:** The inhibition rates of synthesized compounds against NA from H5N1^a^ subtype.

Compounds	10 μM	100 μM	Compounds	10 μM	100 μM
**4a**	73.9%	91.8%	**4h**	40.4%	79.2%
**4b**	50.3%	85.1%	**4i**	32.0%	70.1%
**4c**	43.2%	56.5%	**4j**	20.3%	33.8%
**4d**	30.3%	53.5%	**4k**	0.5%	38.9%
**4e**	28.5%	50.5%	**6i**	63.8%	86.6%
**4f**	24.8%	49.7%	**6j**	37.3%	52.7%
**4g**	ND^b^	ND	**6k**	ND	12.5%
OC	91.0%	95.0%			

^a^ A/Anhui/2005(H5N1). ^b^ Not Determined.

**Table 2:** The IC_50_ values^a^ of several compounds against neuraminidase from H5N1^b^ subtype.

Compounds	OC	4a	4h	4i	6i
IC_50_/μM	0.21 ± 0.021	3.50 ± 0.17	12.00 ± 2.49	20.74 ± 1.14	8.50 ± 0.63

^a^ IC_50_ is compound concentration causing 50% inhibition of NA based on the inhibition rates of 7 different concentrations, values are the mean of three independent experiments. ^b^ A/Anhui/2005(H5N1).

**Table 3:** Human liver microsomal stability of compound **4a**.

Compounds	Microsomal Stability T_1/2_ (min)	Remaining (T = 60 min)
**4a**	>145	101.6%
Testosterone	12.5	3.8%
Diclofenac	9.2	1.1%
Propafenone	5.7	0.1%

## Supplementary Materials

The supplementary materials (Figures S1–S28) are available online: the spectrum of compounds **4a**–**4k** and **6i**–**6k**.

## Abbreviations

NA	neuraminidase
NAs	neuraminidases
NAIs	neuraminidase inhibitors
OC	oseltamivir carboxylate
TEA	triethylamine
