# Supplementary material for: Synthesis and Biological Evaluation of NH2-Sulfonyl Oseltamivir Analogues as Influenza Neuraminidase Inhibitors
Source: Molecules. 2019 Jun 10;24(11):2176. doi: 10.3390/molecules24112176 (PMC6600469; doi:10.3390/molecules24112176)
Supplement: Supplementary file 1 [file molecules-24-02176-s001.pdf]

Supporting information

Synthesis and Biological Evaluation of *NH*$_2$-Sulfonyl Oseltamivir Analogues as Influenza Neuraminidase Inhibitors

Yaping Hu[1], Binfeng Chen[1], Zaiqiang Lei[1], Hongqian Zhao[1], Hongxi Zhu[1], Peng Quan[2,*], Yongshou Tian[1,*]

[1] *Key Laboratory of Structure-Based Drug Design and Discovery, Ministry of Education, School of Pharmaceutical Engineering, Shenyang Pharmaceutical University, Shenyang 110016, Liaoning, China; hyp910309@163.com (Y.H); yh_cbf@163.com (B.C); ZaiqiangLei@126.com (Z.L); zhaohongqian999@163.com (H.Z); 13125557362@163.com (H.Z.)*

[2] *School of Pharmacy, Shenyang Pharmaceutical University, Shenyang 110016, Liaoning, China*

* Correspondence: quanpeng81@163.com (P.Q.); tianys@syphu.edu.cn (Y.T.);

Tel.: +86-24-4352-0511 (P.Q.); +86-24-4352-0511 (Y.T.)

The copies of spectrum for compounds 4a-4k and 6i-6k.

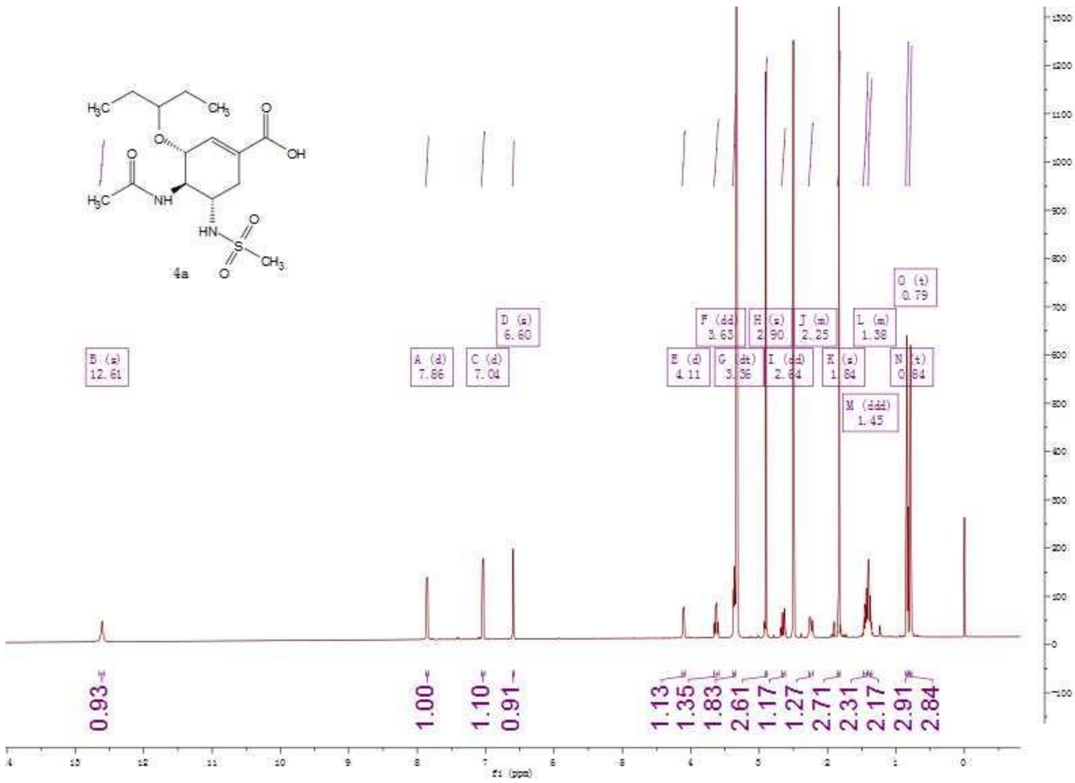

Figure S1. ^{1}H-NMR of compound **4a**.

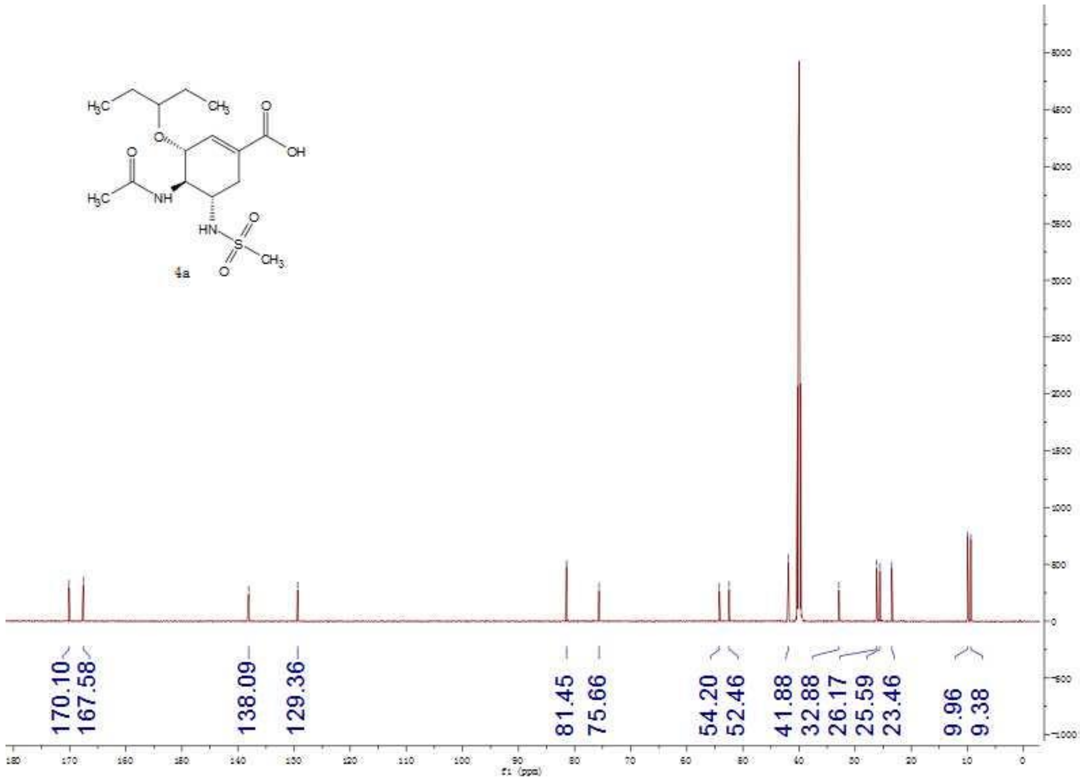

Figure S2. ^{13}C-NMR of compound **4a**.

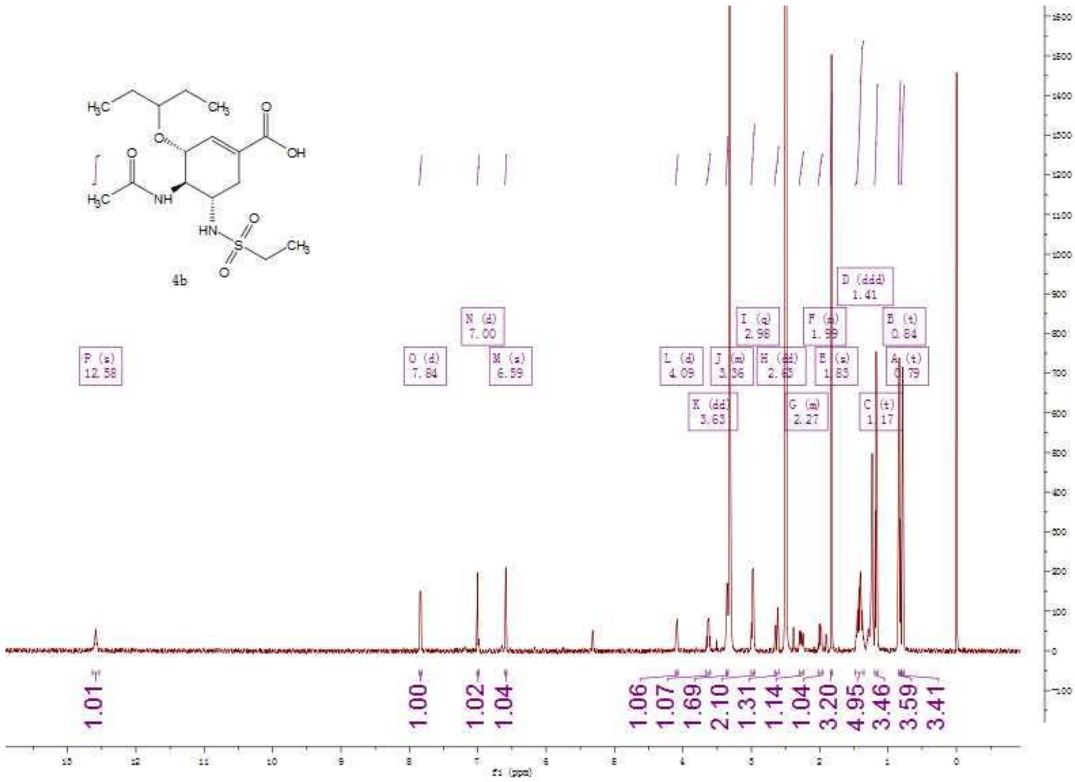

Figure S3. ^{1}H-NMR of compound **4b**.

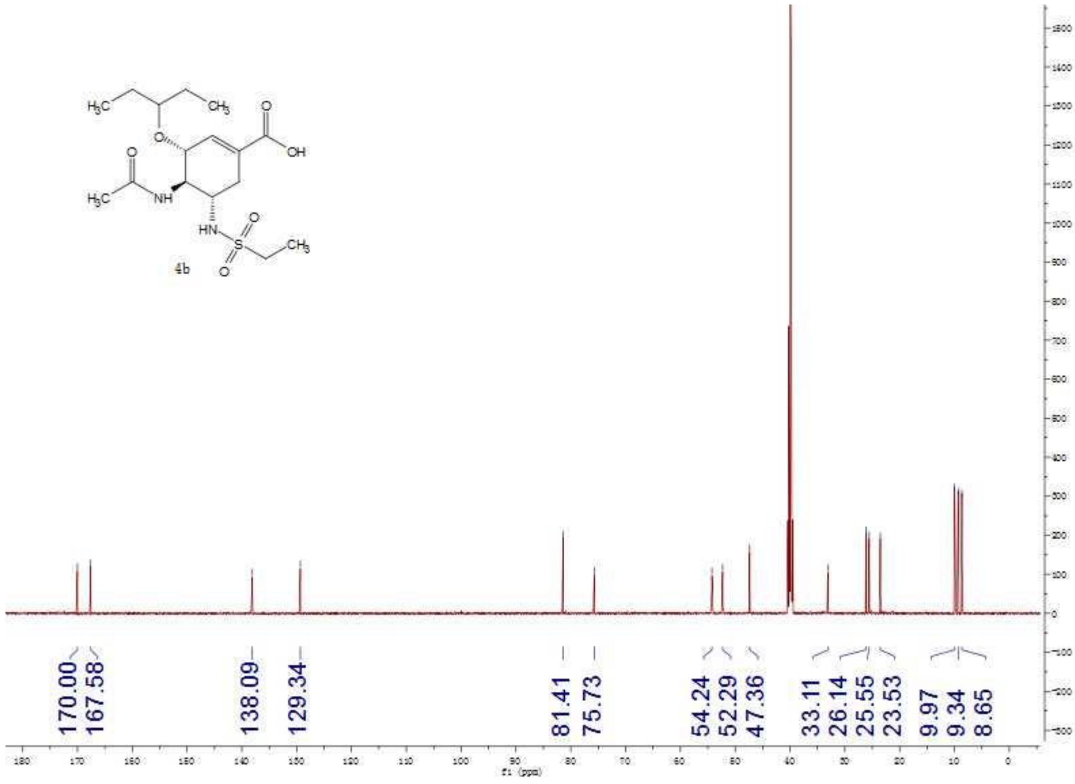

Figure S4. ^{13}C-NMR of compound **4b**.

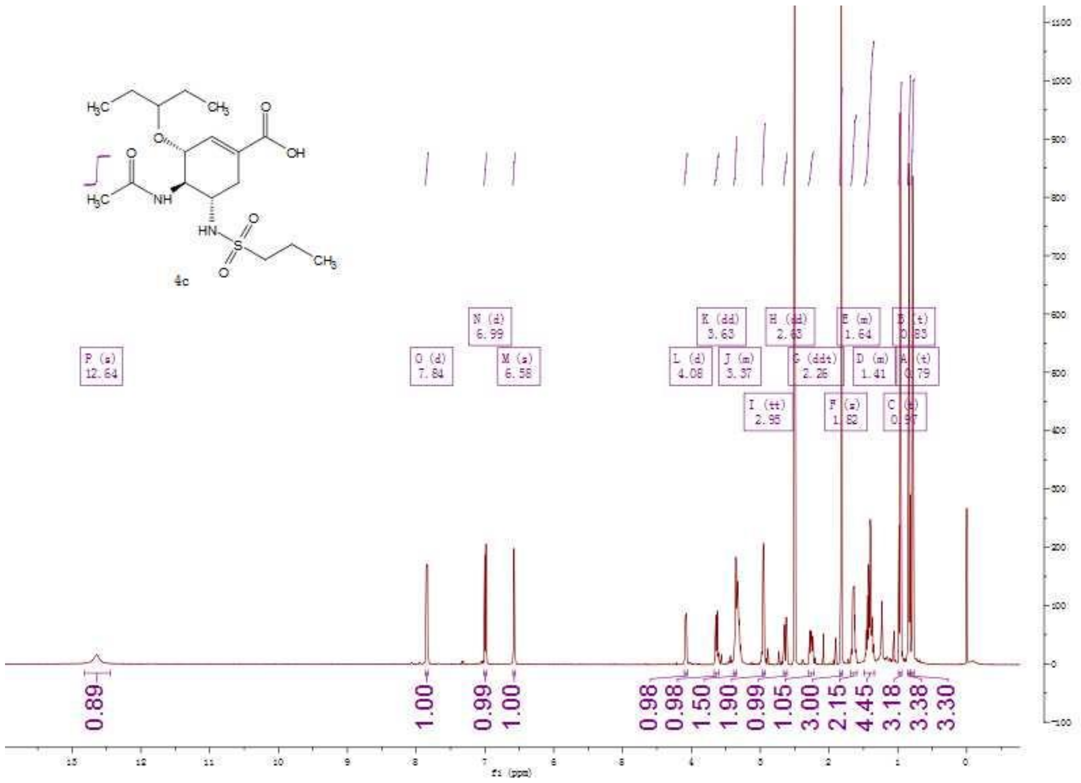

Figure S5. ^{1}H-NMR of compound **4c**.

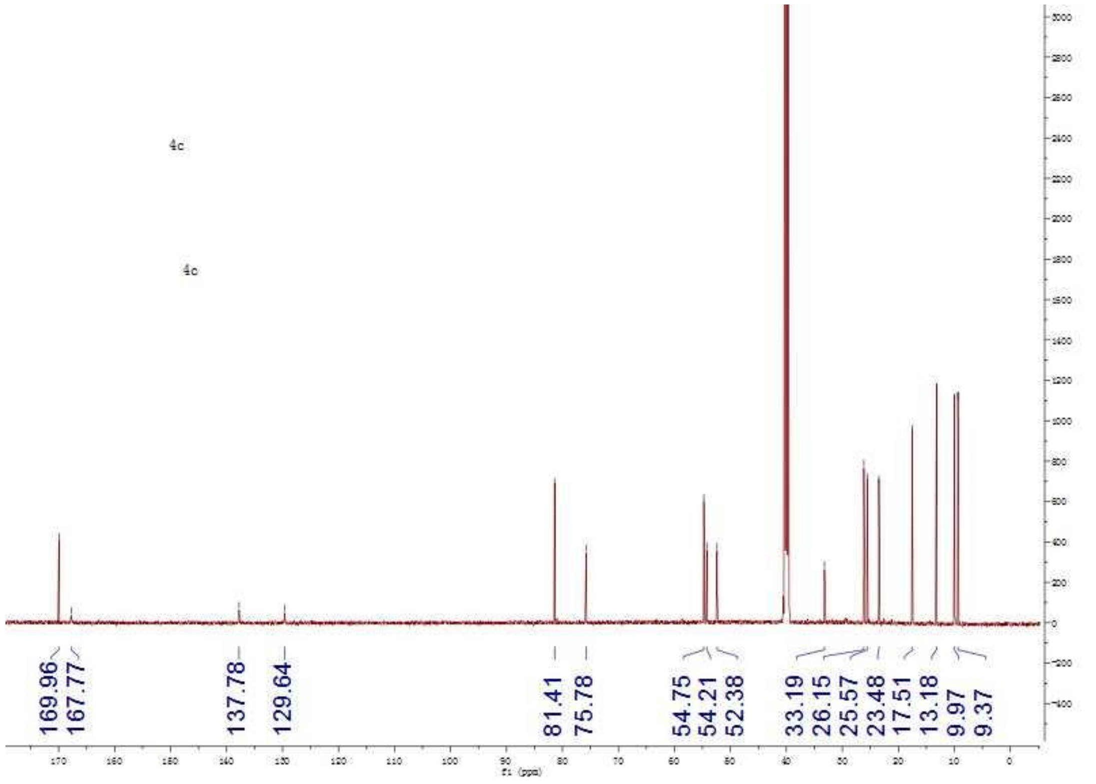

Figure S6. ^{13}C-NMR of compound **4c**.

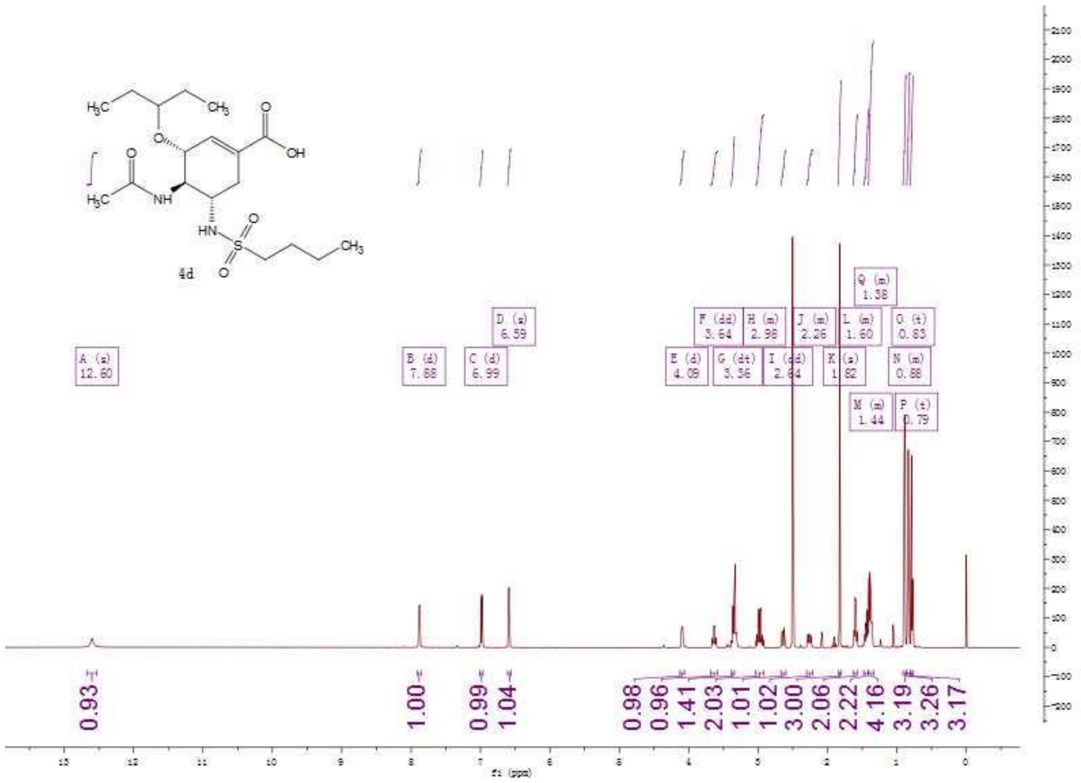

Figure S7. ^{1}H-NMR of compound **4d**.

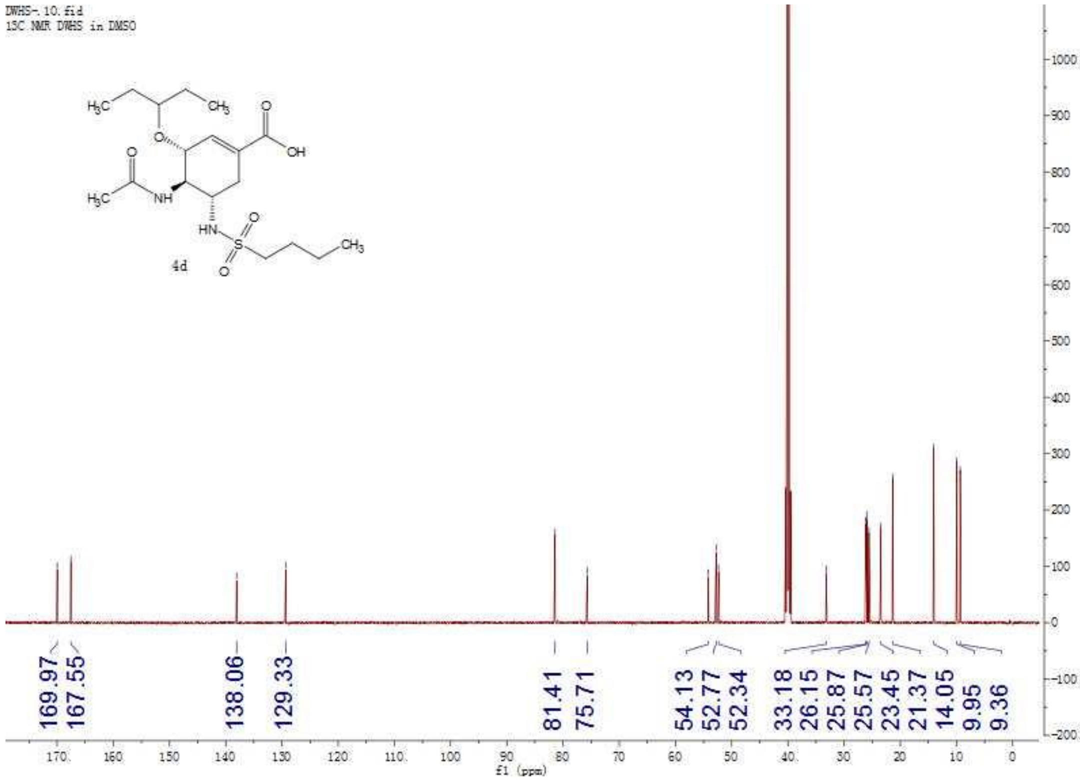

Figure S8. ^{13}C-NMR of compound **4d**.

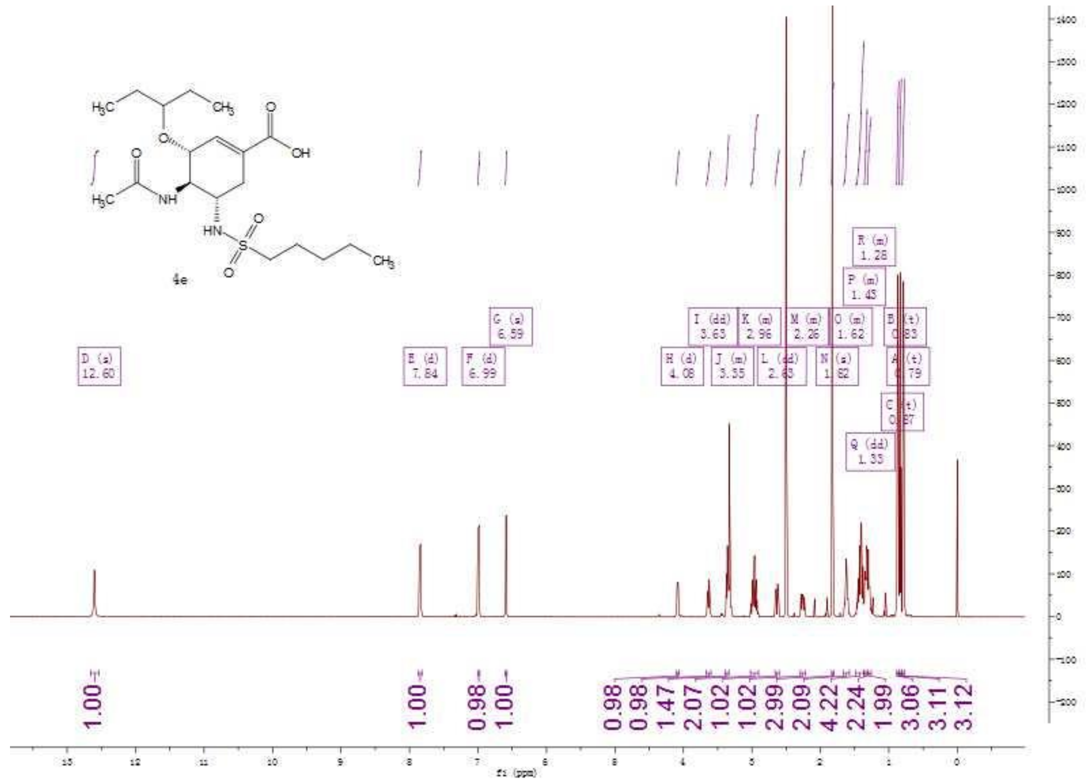

Figure S9. ^{1}H-NMR of compound **4e**.

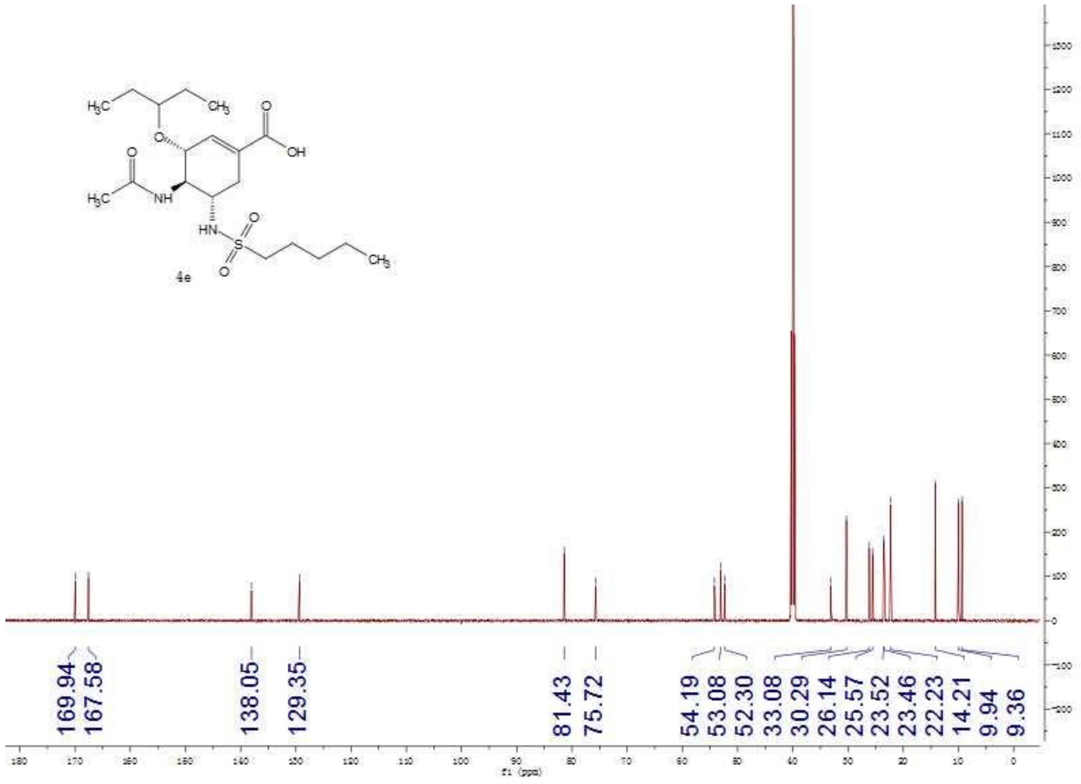

Figure S10. ^{13}C-NMR of compound **4e**.

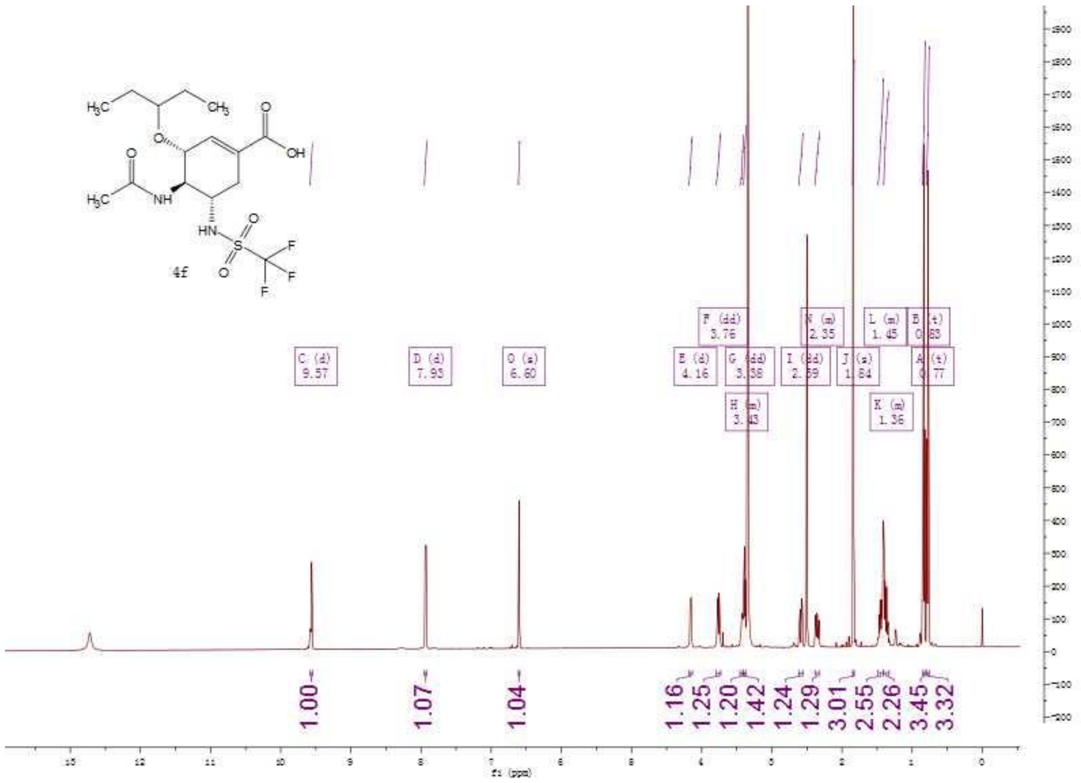

Figure S11. ^{1}H-NMR of compound **4f**.

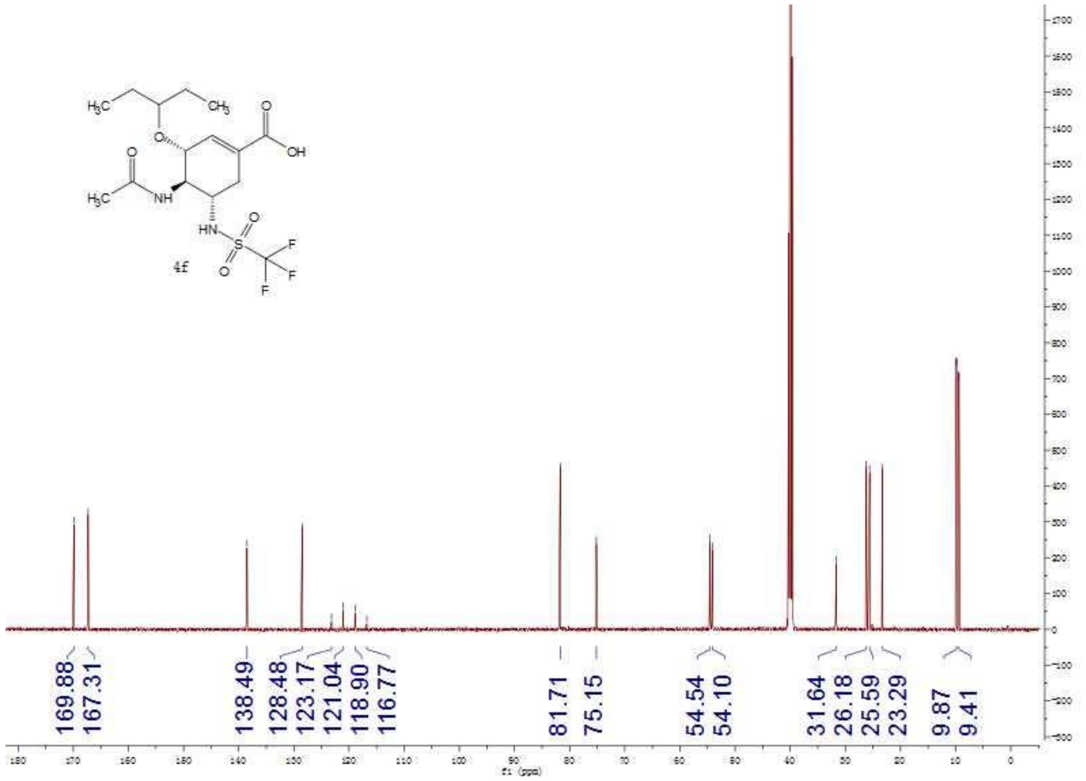

Figure S12. ^{13}C-NMR of compound **4f**.

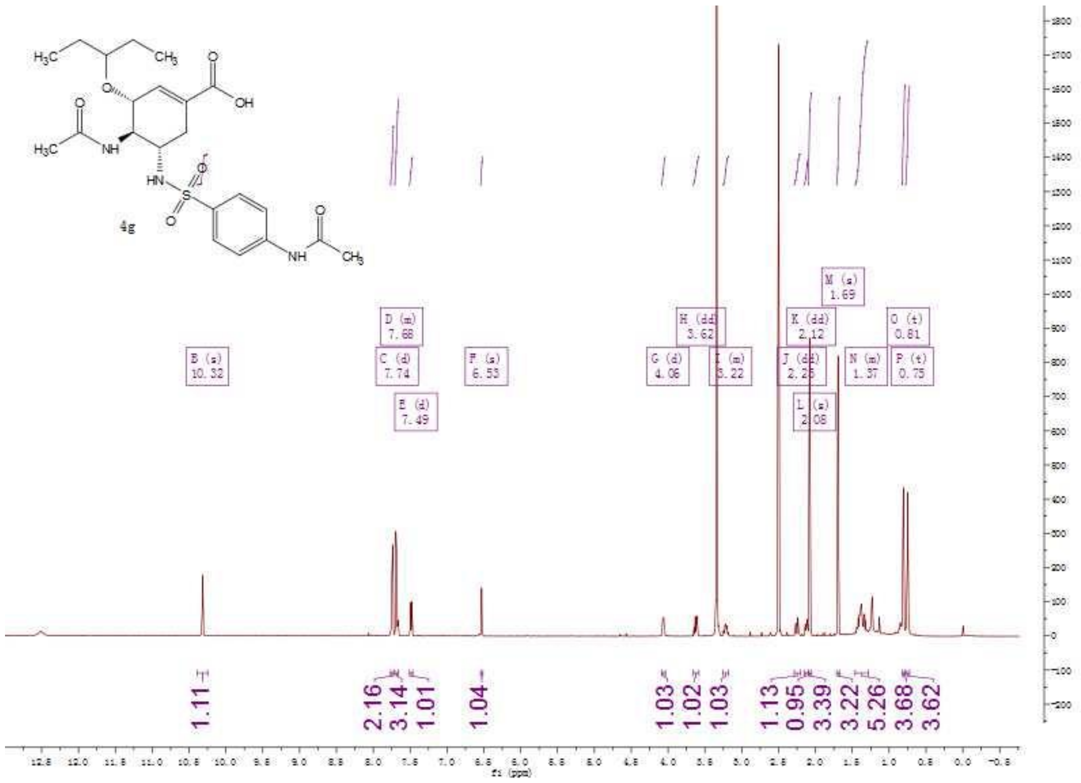

Figure S13. ^{1}H-NMR of compound **4g**.

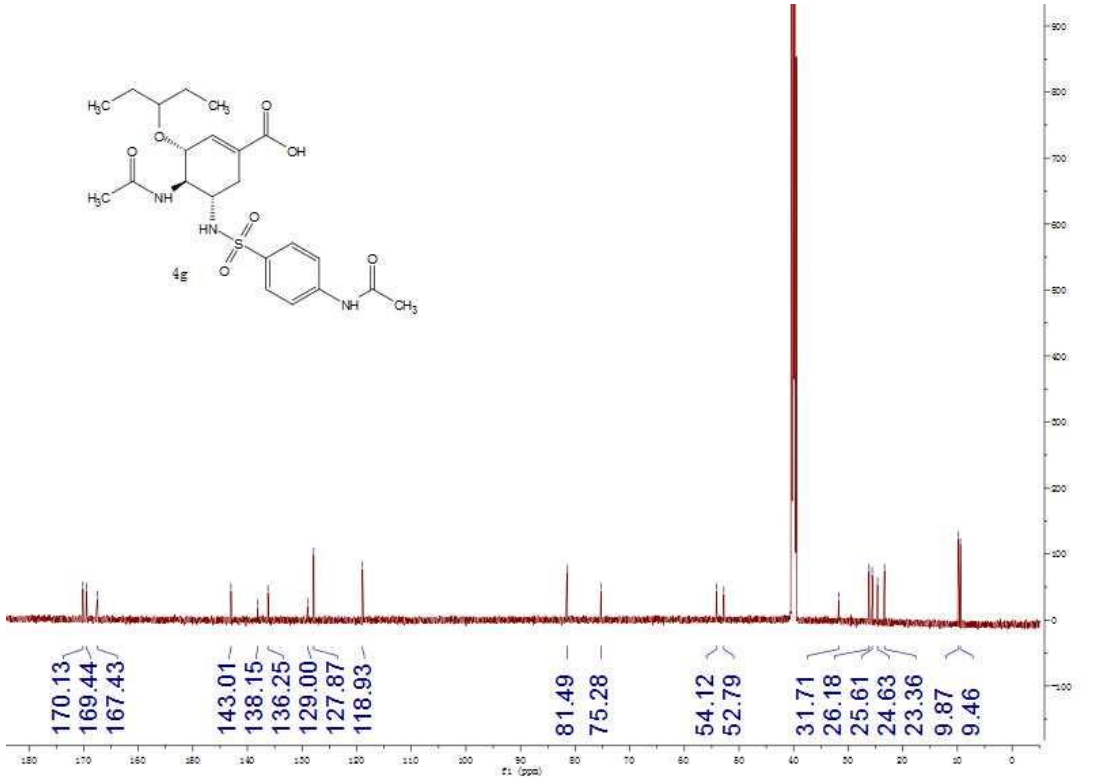

Figure S14. ^{13}C-NMR of compound **4g**.

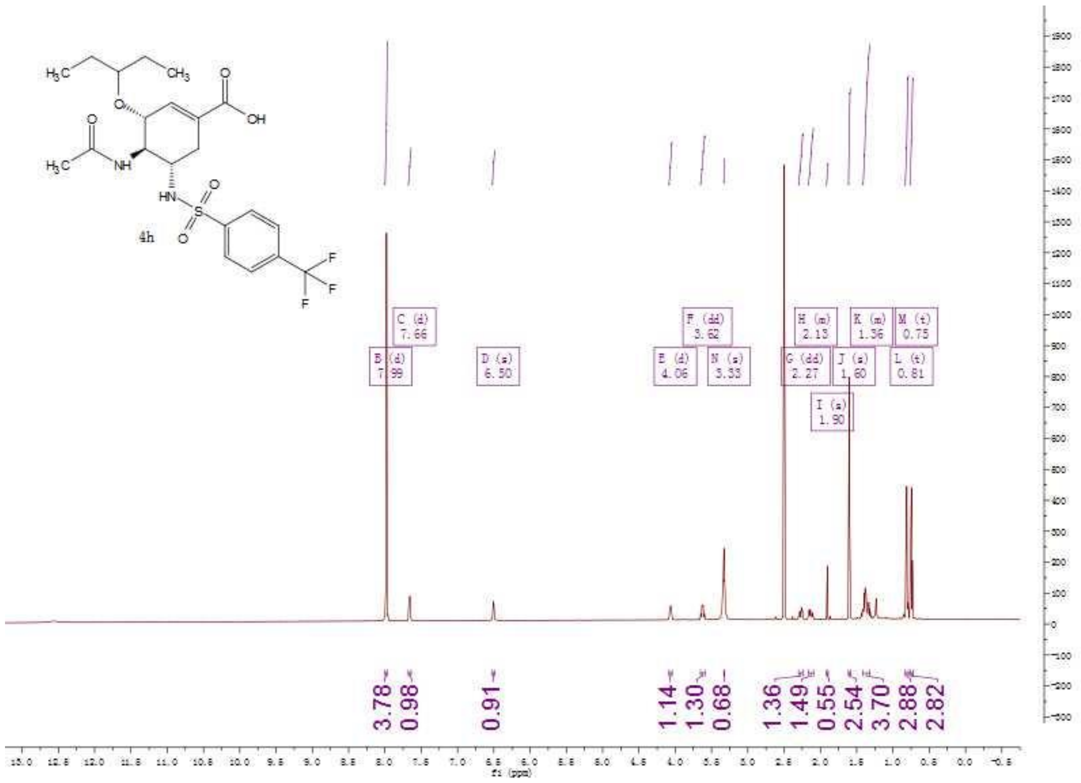

Figure S15. ^{1}H-NMR of compound **4h**.

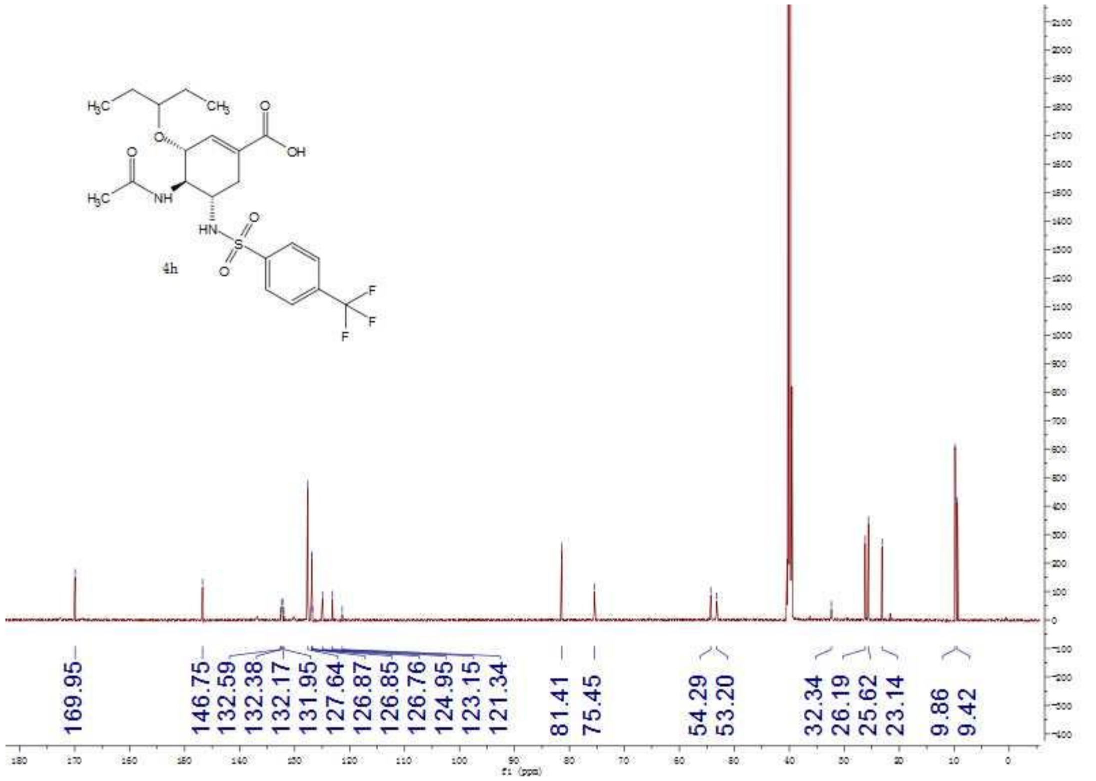

Figure S16. ^{13}C-NMR of compound **4h**.

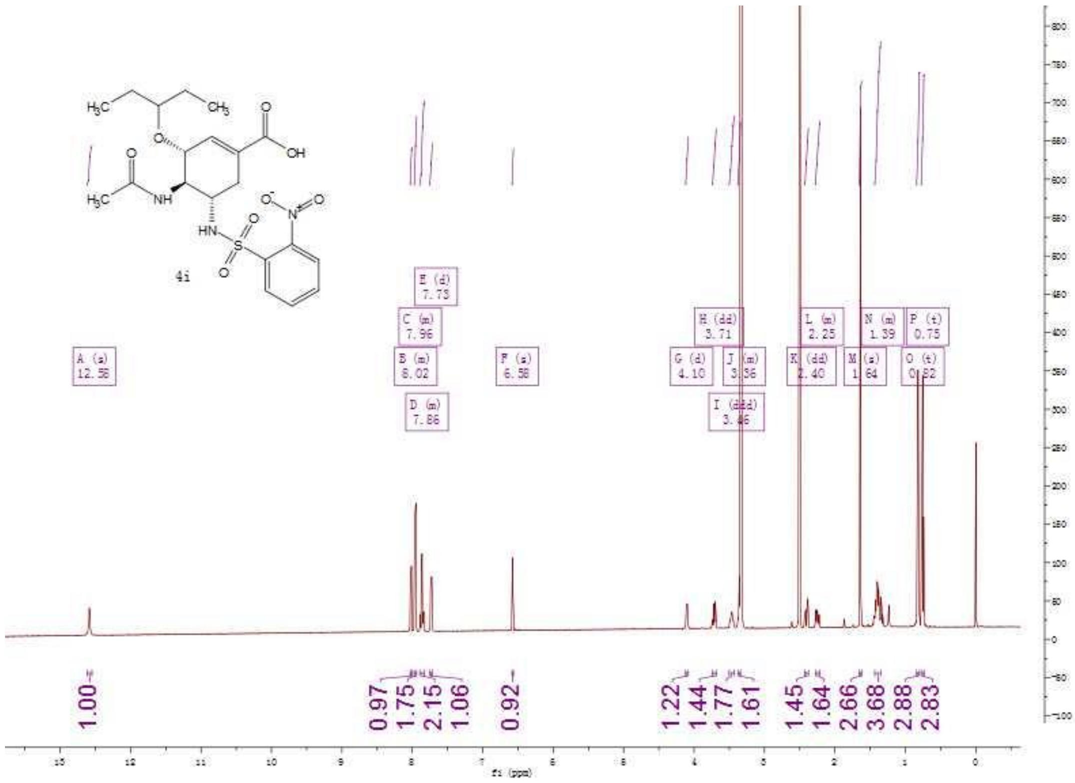

Figure S17. ^{1}H-NMR of compound **4i**.

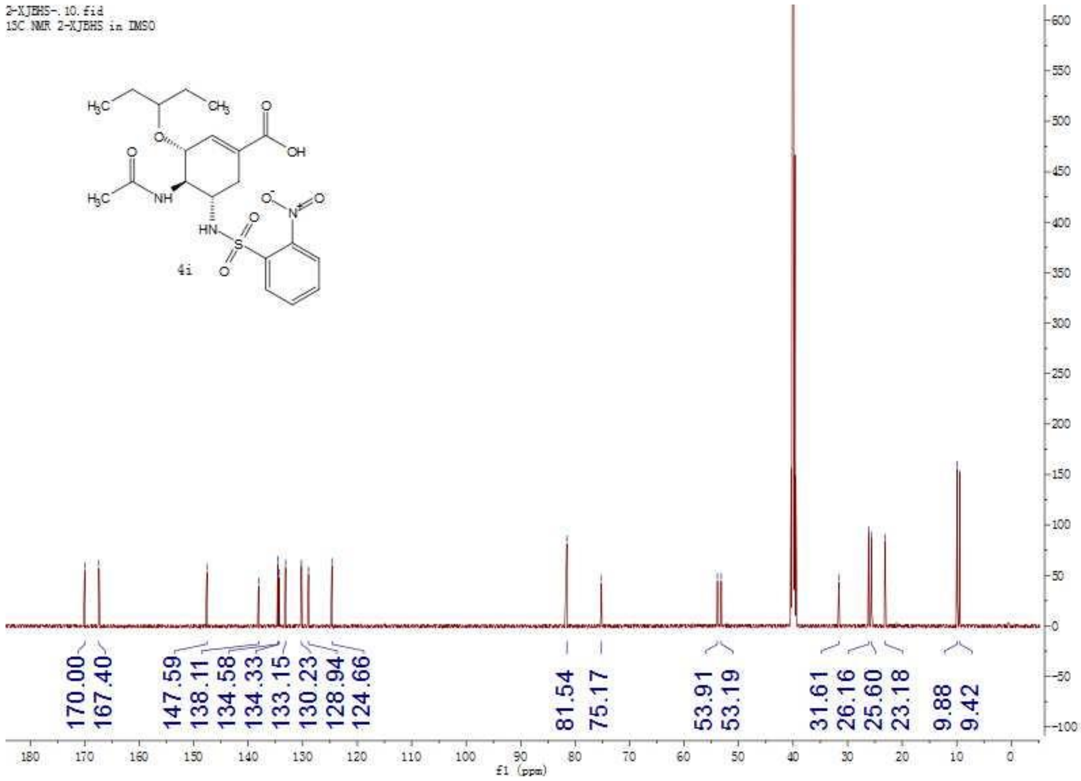

Figure S18. ^{13}C-NMR of compound **4i**.

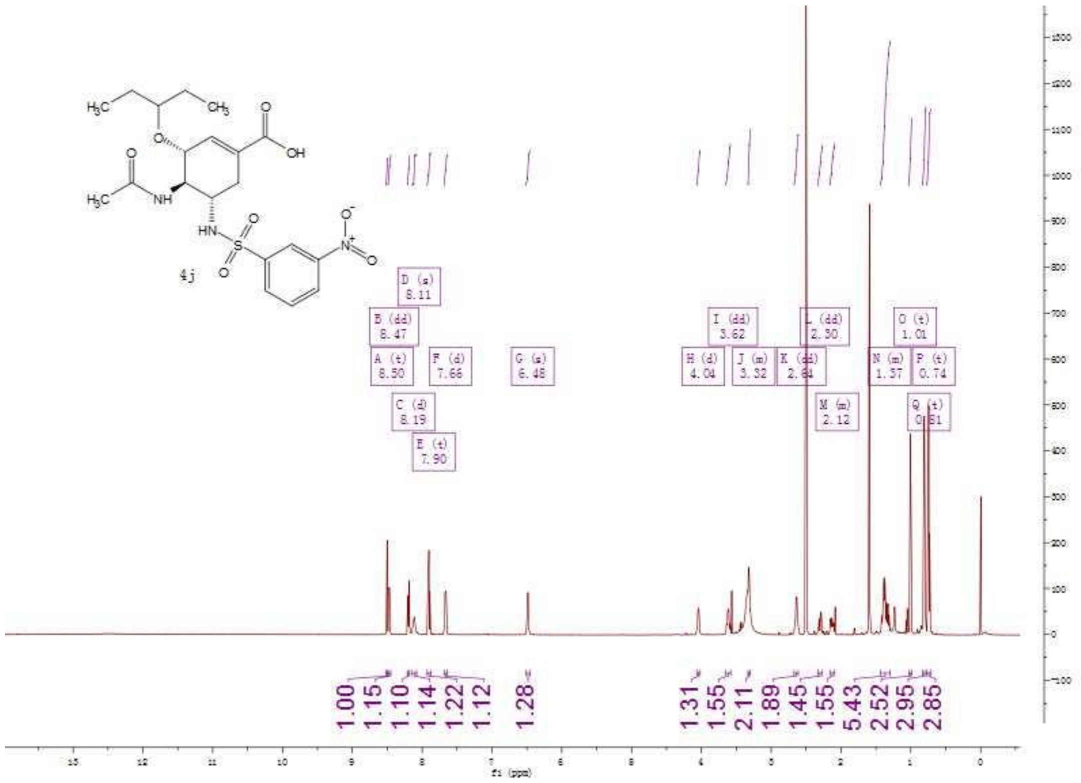

Figure S19. ^{1}H-NMR of compound **4j.**

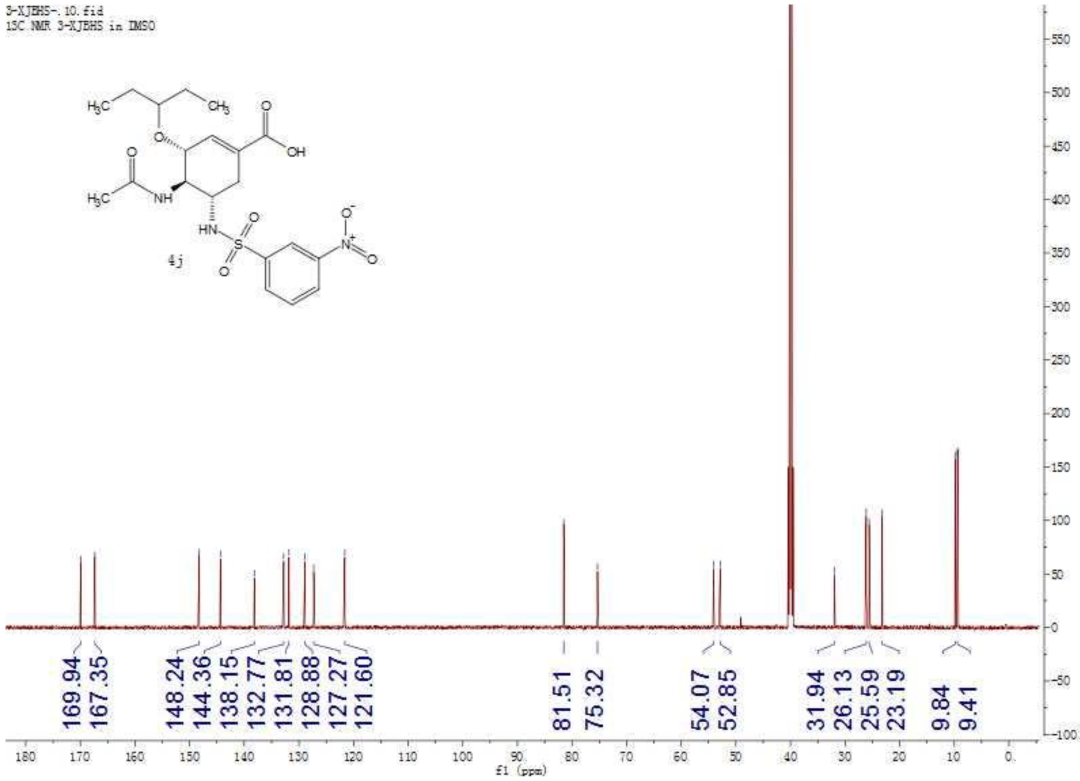

Figure S20. ^{13}C-NMR of compound **4j**.

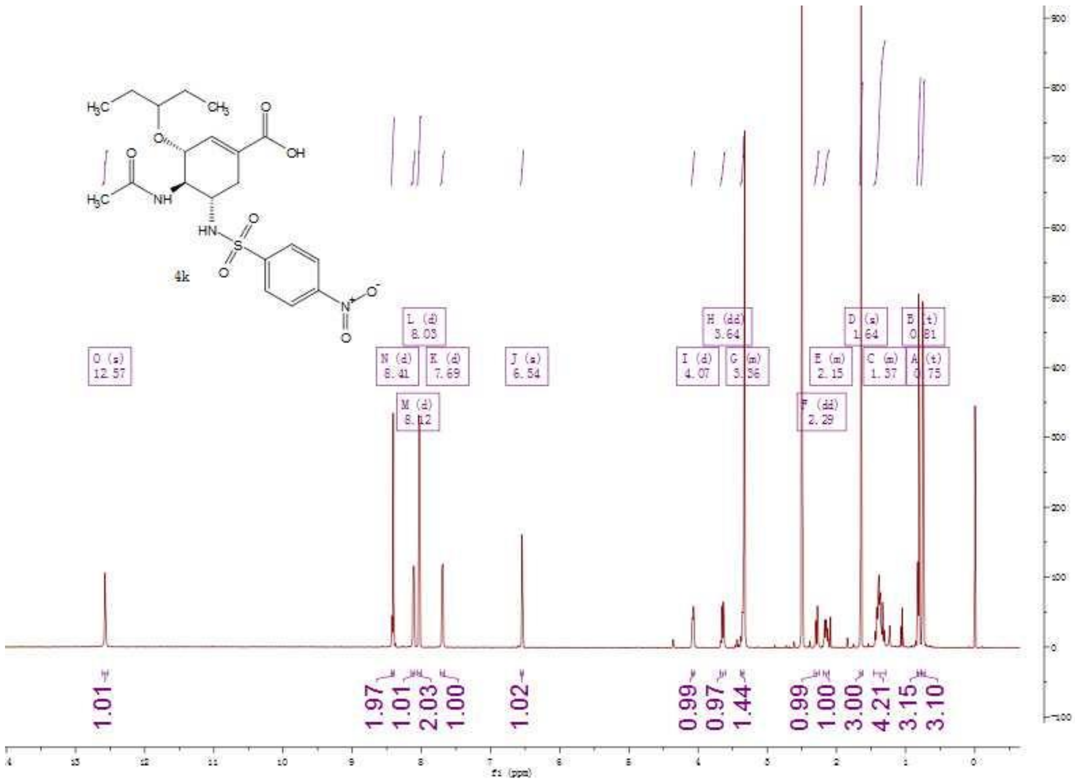

Figure S21. ^{1}H-NMR of compound **4k**.

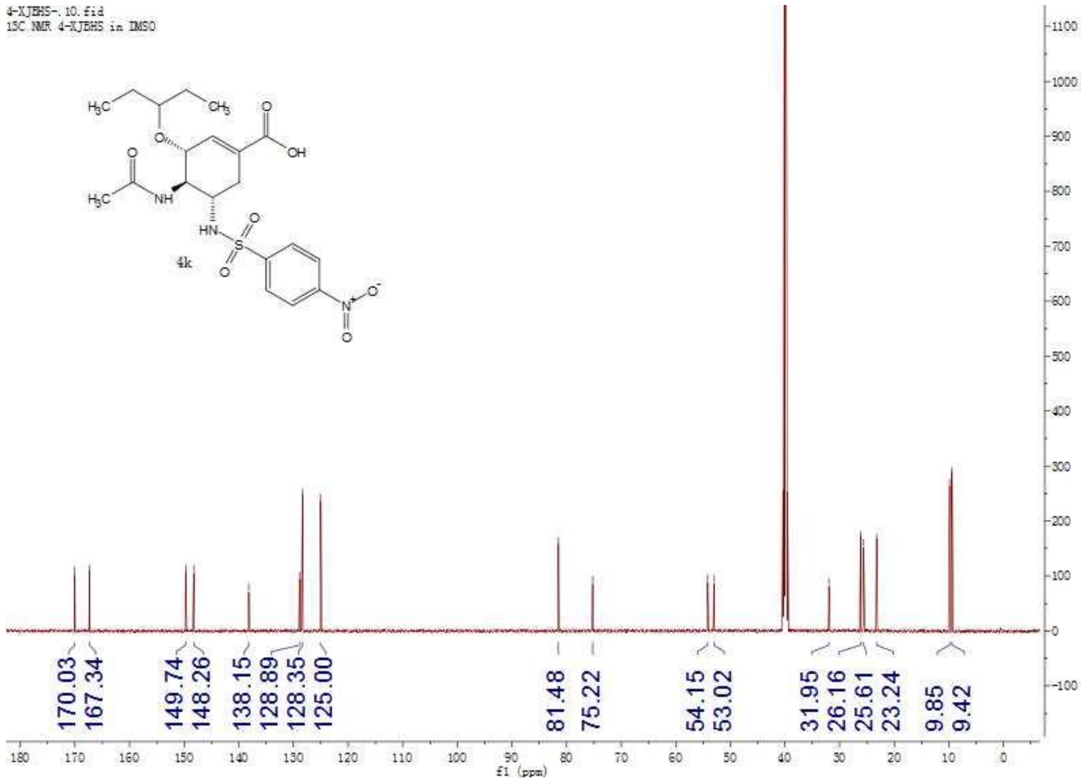

Figure S22. ^{13}C-NMR of compound **4k**.

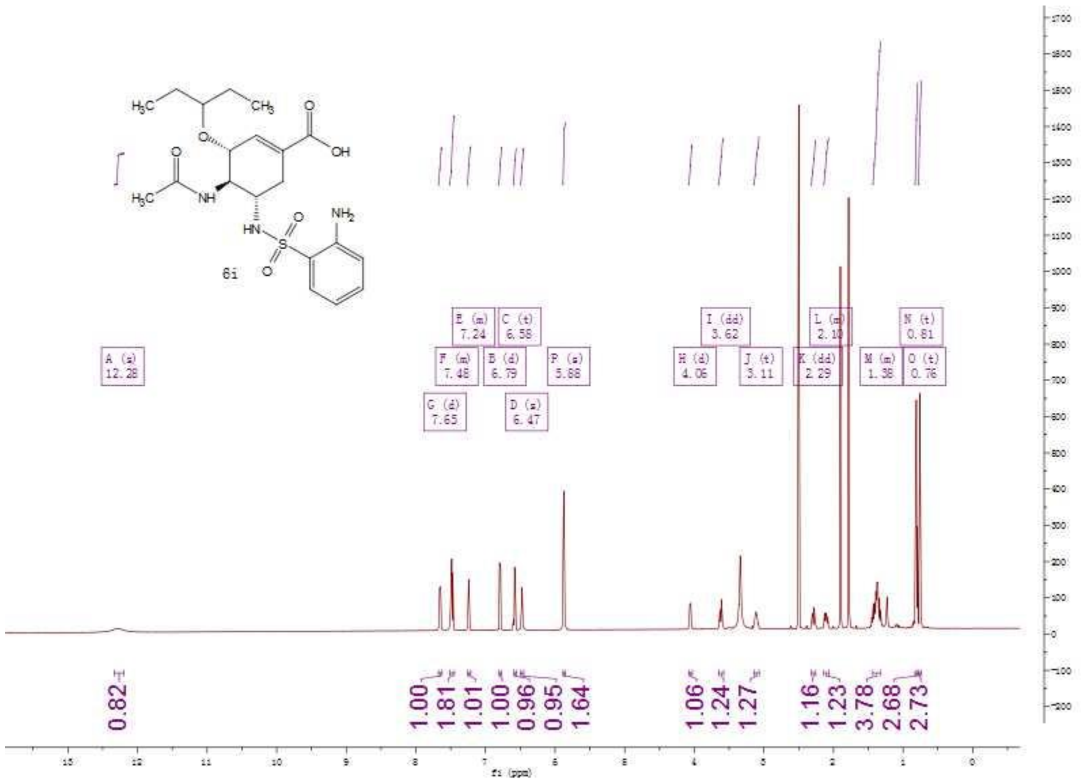

Figure S23. ^{1}H-NMR of compound **6i**.

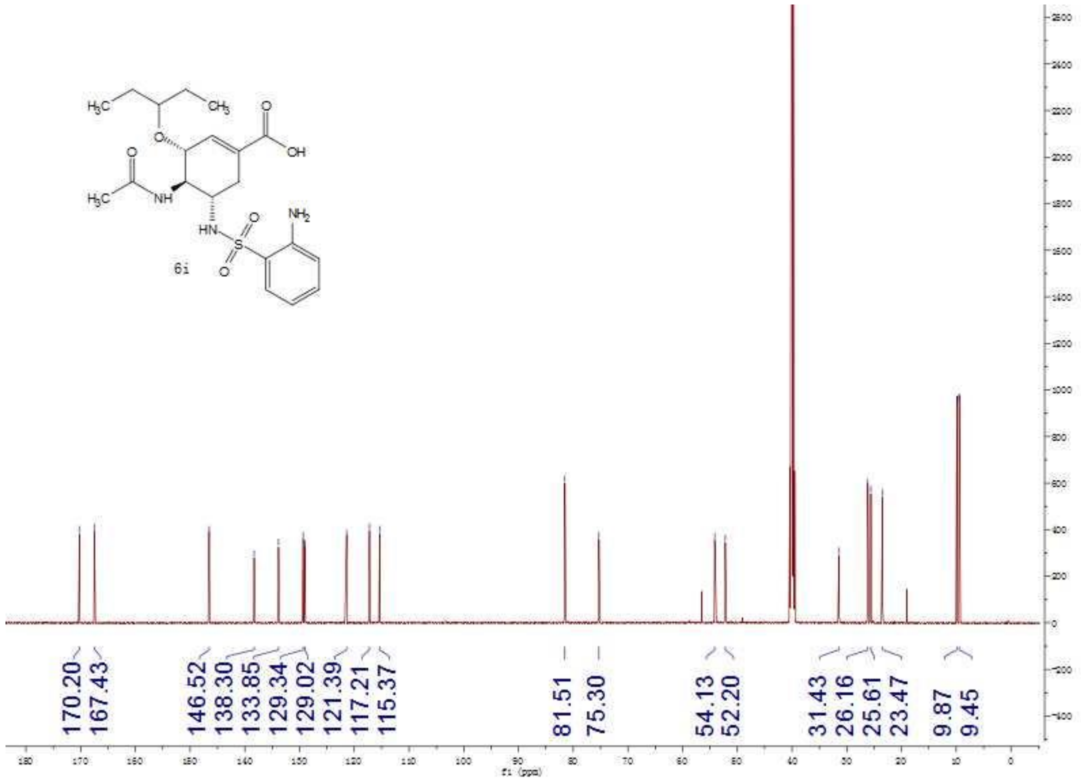

Figure S24. ^{13}C-NMR of compound **6i**.

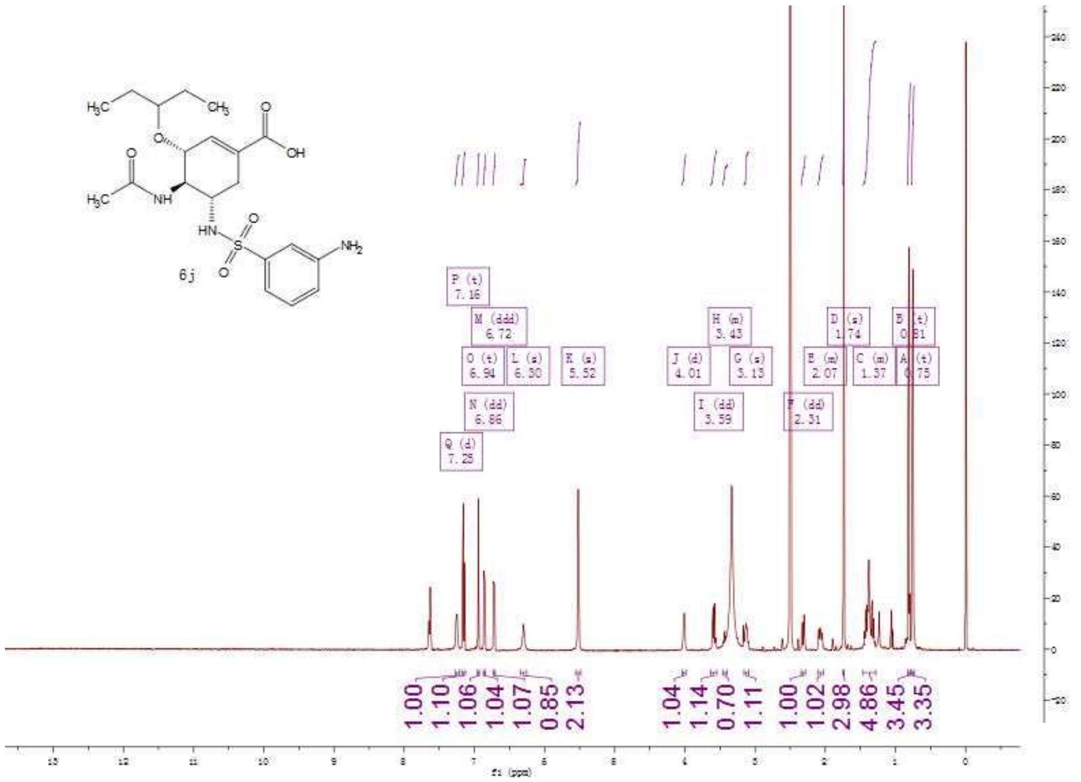

Figure S25. ^{1}H-NMR of compound **6j**.

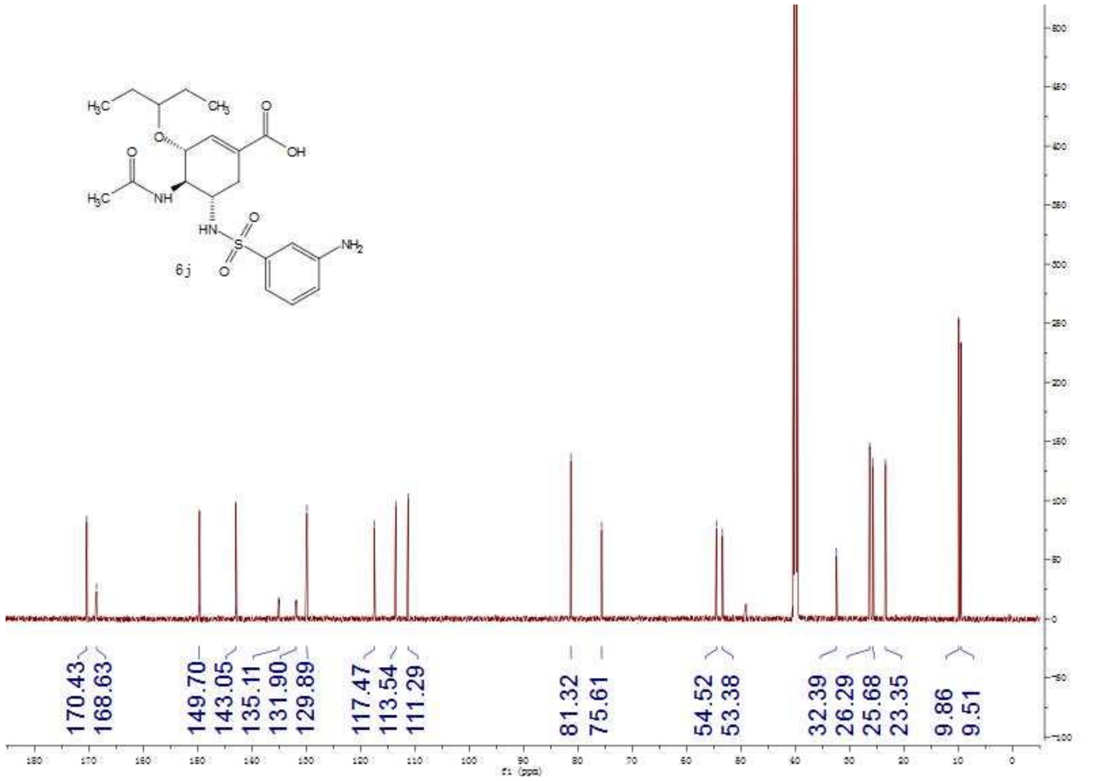

Figure S26. ^{13}C-NMR of compound **6j**.

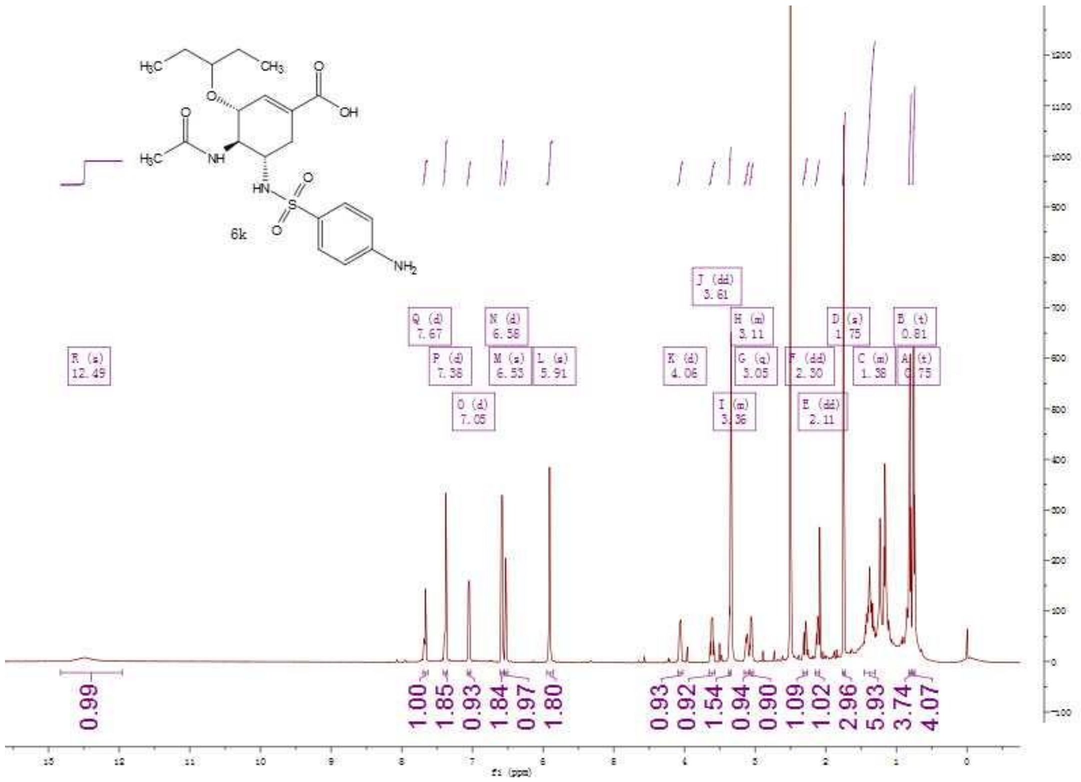

Figure S27. ^{1}H-NMR of compound **6k**.

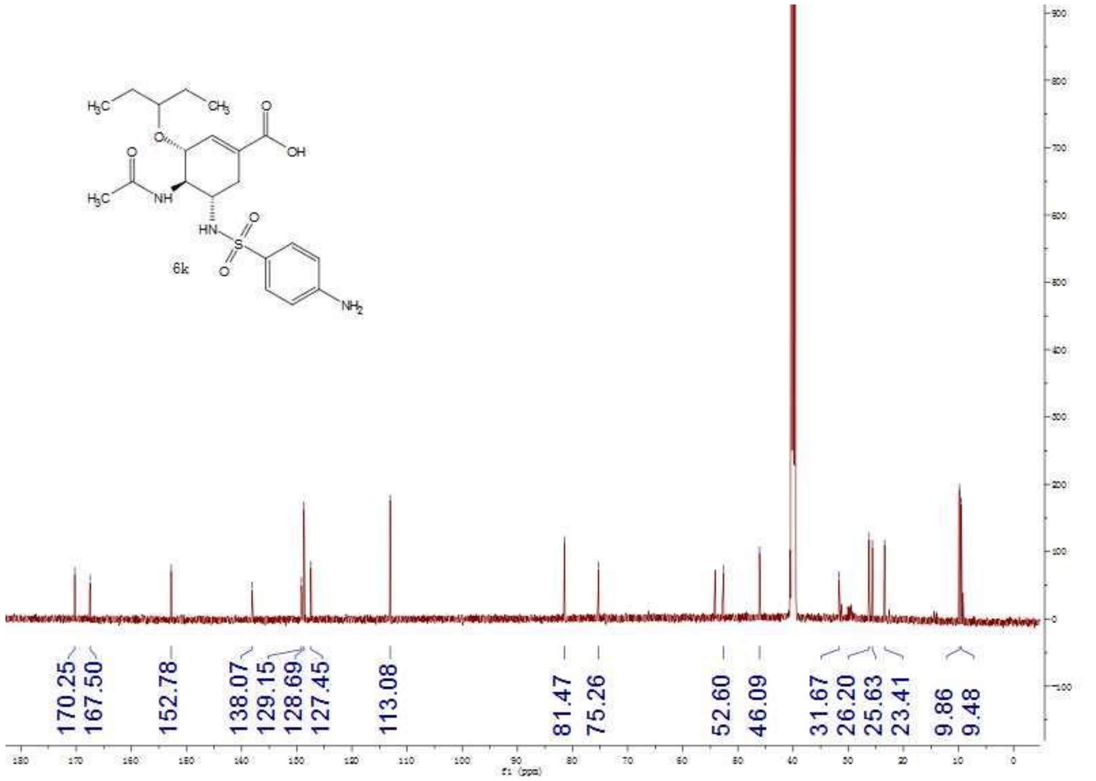

Figure S28. ^{13}C-NMR of compound **6k**.
